# A Broader Survey on 6G Radio Resource Management

**DOI:** 10.3390/s26082497

**Published:** 2026-04-17

**Authors:** Afonso José de Faria, José Marcos Câmara Brito, Danilo Henrique Spadoti, Ramon Maia Borges

**Affiliations:** 1 Institute of Systems Engineering and Information Technology, Federal University of Itajubá, Itajubá 37500-903, MG, Brazilspadoti@unifei.edu.br (D.H.S.); ramonmb@unifei.edu.br (R.M.B.); 2 Instituto Nacional de Telecomunicações, Santa Rita do Sapucaí 37536-001, MG, Brazil

**Keywords:** 6G, cell-free, energy efficiency, heterogeneous networks, radio resource management, spectral efficiency

## Abstract

The sixth-generation (6G) mobile communication systems are anticipated to be operational by 2030, prompting extensive research efforts by governments and private entities. Designed to meet societal, economic, and technological demands unaddressed by fifth-generation (5G) networks, 6G integrates scalability, security, and reliability with ubiquity and resource-intensive artificial intelligence. Envisaged as multi-band, decentralized, autonomous, flexible, and user-centric, 6G networks incorporate innovative technologies, including cell-free (CF), three-dimensional heterogeneous networks (3D HetNet), reconfigurable intelligent surfaces (RIS), integrated sensing and communication (ISAC), as well as artificial intelligence/machine learning (ML). In 6G 3D HetNets, the densification of access points (APs) continues, accommodating increased connections and traffic volumes, alongside the use of higher frequency bands. Although 6G networks are not fully standardized, they target demanding Quality of Service (QoS) standards, such as a peak data rate of 1.0 Tbps and latency of 0.1 ms. This paper conducts a comprehensive literature review on radio resource management (RRM) in 6G cell-free and 3D HetNet systems, emphasizing challenges such as interference mitigation. It presents a taxonomy of RRM approaches, systematically studying, categorizing, and qualitatively analyzing recent techniques, outlining the current state, and indicating future trends, technologies, and challenges shaping 6G systems.

## 1. Introduction

The sixth-generation (6G) mobile communication system is expected to build on the advances of fifth-generation (5G) while introducing capabilities to deliver enhanced and new value-added services. Key priorities include promoting digital inclusion, ensuring environmental and economic sustainability, managing traffic growth, and enabling applications through features that complement existing mobile networks. Thus, both academia and industry are investigating enabling technologies to shape 6G systems characterized by intelligent, green, flexible, and secure paradigms and architecture [1,2,3].

The study of radio resource management (RRM) in the 6G ecosystem follows a well-established industry trend of periodically reviewing mobile network capabilities to address societal and user needs. With new mobile generations typically emerging every decade and the latest milestone being International Mobile Telecommunications (IMT)-2020, it is now time to consider the connectivity requirements for IMT-2030 and beyond [4].

IMT-2030 is expected to integrate innovative technical solutions toward a network infrastructure able to support 6G trends [3,4,5]:

- Ubiquitous Intelligence and Computing: With advancements in artificial intelligence (AI) and machine learning (ML), it is expected that intelligence will be embedded throughout the communication system, facilitating smart cities and communities. Devices will become context-aware, enhancing interactions between humans, machines, and the environment. AI and ML may enable autonomous network management, including self-monitoring, self-organization, self-optimization, and self-healing capabilities.
- Immersive Multimedia and Interactions: IMT-2030 will likely transform multimedia and human-centric communication, offering immersive experiences through multi-sensory interactions. This includes personalized extended reality, holographic telepresence for various activities, and new human–machine interfaces that enable remote operations and interactions. The integration of edge cloud computing and AI will support tactile internet and ambient awareness.
- Smart Industrial Applications: IMT-2030 will support smart industrial applications by enabling real-time intelligence exchange, resource optimization, and automated processes. Reliable and low-latency connectivity, along with precise environmental awareness, will be crucial for applications such as manufacturing optimization, automated product delivery, and high-precision positioning.
- Reimagined Network Architecture and Devices: The 6G era will introduce new network architecture paradigms, characterized by an open, scalable, secure, and distributed heterogeneous cloud system. The latter one will include hardware acceleration options and a common framework for radio access network (RAN) and core networks to reduce complexity. The network is expected to support dynamic offloading, flexible instantiation of sub-networks and AI-powered programmability. The ubiquity and longevity of Internet of Things (IoT) devices will be enhanced through zero-energy devices, furthering their deployment and utility.
- Network of Networks: 6G will encompass multiple physical and virtual sub-networks. The evolution of private networks will support machines and processes demanding stringent quality-of-service (QoS). Verticals and enterprises will benefit from automated services with guaranteed performance, facilitated by an as-a-service business model that integrates communication, data, and AI resources to enable new value chains and customized network capabilities.
- Integrated Sensing and Communication (ISAC) and Native AI: Balancing communication metrics with sensing accuracy and AI-driven decisions in real time is expected to contribute to ubiquitous connectivity, important for delivering services in education, health, agriculture, transport, logistics, and business.
- Sustainability: Sustainability is a core goal for IMT-2030, aiming to meet environmental, social, and economic targets. Future networks will focus on minimizing environmental impact through efficient resource use, reducing power consumption, and leveraging circular economy principles. A wide range of sustainability aspects must be considered, as outlined by the United Nations Sustainable Development Goals (SDGs), particularly SDG 11 (sustainable cities and communities) and SDG 13 (climate action). High energy efficiency can directly translate to lower carbon dioxide (CO_2_) emissions. In this way, key performance indicators (KPIs) for 6G include energy efficiency, reinforcing the centrality of sustainability in future networks.

### 1.1. Motivation

The vision for 6G networks is to create a hyper-connected world where seamless and ubiquitous connectivity powers the next generation of applications and services. Such vision is reflected in stringent KPIs set by standardization bodies like the International Telecommunication Union (ITU) and specifications from organizations like the 3rd Generation Partnership Project (3GPP). These KPIs encompass aspects such as network capacity, user experience (latency, data rates), energy efficiency and security. However, efficiently managing resources and meeting 6G KPIs presents significant challenges compared to previous generations, e.g., due to the extreme network densification, the dynamic traffic patterns, and the expected integration of Terrestrial Networks (TN) and Non-Terrestrial Networks (NTN) toward coverage gains.

The challenges in 6G RRM can be categorized into three main aspects:

- Network Complexity: Emerging technologies like cell-free (CF) networks and three-dimensional (3D) heterogeneous deployments introduce new complexities in resource allocation and interference management.
- Diverse User Needs: Catering to a wider range of devices with varying data rate and latency requirements demands flexible resource allocation strategies.
- Dynamic Network Environment: Rapidly changing traffic patterns and user locations require real-time resource allocation adjustments to maintain optimal performance.

This work is motivated by the escalating difficulty of RRM in the face of extreme network densification and the integration of heterogeneous 3D architectures, including TN and NTN. Solving optimization problems for resource allocation and scheduling demands sophisticated RRM strategies and is valuable for realizing the ambitious goals of 6G. The current study addresses these problems, including the objective functions, constraints, and methodologies used for optimization. Additionally, key enabling technologies for efficient 6G RRM are explored, such as massive multiple-input multiple-output (mMIMO), beamforming, non-orthogonal multiple access (NOMA), intelligent reflecting surfaces (IRS), terahertz (THz) spectrum, AI, and advanced sensing techniques. These technologies offer promising solutions to navigate the complexities of 6G RRM. Questions that guide the study include: “What are the challenges and trends in 6G radio resource management?”; “How to solve optimization problems for 6G resource allocation and scheduling?”; “What are the key enabling technologies for efficient radio resource management in 6G?”; “What are the future research opportunities for 6G?”

Strategically, advancing 6G RRM research and development may enhance the overall performance and capabilities of 6G networks. Achieving efficient RRM can lead to better utilization of network resources, improved QoS for end-users, and greater operational efficiency for service providers. The impact of these advancements may be far-reaching, enabling a new era of connectivity that supports transformative applications and services across various industries.

### 1.2. Contributions

Surveys addressing 6G have been published in the literature with focus on several and relevant topics, such as: advances in THz [6,7], IRS [8,9], ISAC [10,11,12], optical fronthaul [13], open RAN [14], quality of experience (QoE) [15], security [16,17,18], AI- and ML-driven capabilities [19,20,21,22,23], resource management [24,25,26], and others. Notably, many of these studies integrate and emphasize different topics, e.g., the papers [7,9,12,20,23]. Regarding 6G resource management, the authors in [24] focus on the progress of network slicing, paper [25] underscores challenges in sidelink resource allocation, whereas Jaradat et al. report an overview of radio resource scheduling [26].

This work provides a broader and comprehensive overview of RRM for future 6G networks. Based on an extensive review of recent publications and considering the effective application of the cell-free concept in 6G systems and the integration of TN and NTN communication media, this survey makes the following contributions:

- Comprehensive review of enabling technologies: We offer a detailed examination of the promising technologies to ensure the safe and efficient operation of 6G networks, aligned with the envisioned performance indicators.
- Analysis of challenges and trends in 6G RRM: We identify and discuss the primary challenges and emerging trends in 6G radio resource management, highlighting the complexities involved in meeting the stringent requirements of 6G networks.
- Future research opportunities: Recognizing the innovative services and applications anticipated for 6G, as well as the ambitious performance goals, we outline key areas for future research toward effective implementation of 6G networks.
- Strategies and solutions for optimized resource allocation and scheduling: We present an overview of the strategies and solutions proposed in the literature to enhance network performance and resource efficiency. This includes optimized resource allocation and scheduling through the use of innovative algorithms and methods.

The contributions aim to provide a solid foundation for understanding and advancing radio resource management in 6G networks, guiding future research and development efforts in this critical area.

### 1.3. Paper Organization

**Figure 1:**
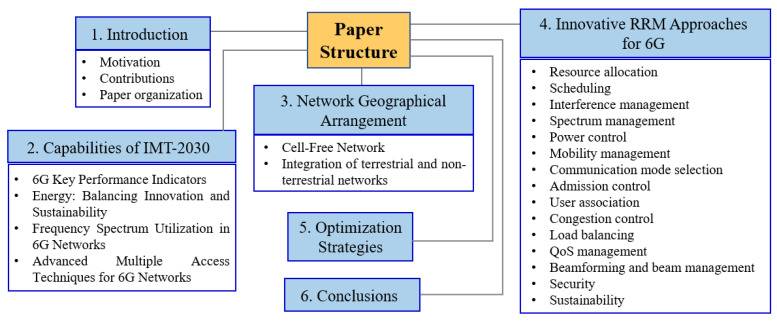
Graphical table of contents.

Figure 1 illustrates the organization of the paper, which is structured as follows:

- Section 1 (Introduction): Provides the context, challenges, and vision for 6G networks, along with the specific research motivations and contributions of the study.
- Section 2 (Capabilities of IMT-2030): Initiates with an overview of the projected standardization and deployment timeline from the perspectives of 3GPP and ITU, next exploring the capabilities of IMT-2030.
- Section 3 (Network Geographical Arrangement): Discusses the 6G network geographical arrangement, analyzing the structure of cellular and cell-free networks. This section also addresses the evolving design of two- and three-dimensional heterogeneous networks (2D and 3D HetNets), which are important for supporting diverse communication environments.
- Section 4 (Innovative RRM Approaches for 6G): Delves into innovative approaches to RRM in 6G systems. It outlines the key RRM functions, including resource allocation, scheduling, interference management, spectrum management, power control, mobility management, and communication mode selection.
- Section 5 (Optimization Strategies): Presents diverse optimization strategies and analytical frameworks designed for resource allocation in 6G RRM. This section also reviews key algorithms, ranging from classical approaches to modern AI-driven solutions, that aim to address these challenges and enhance resource management efficiency.
- Section 6 (Conclusions): Offers the conclusions of this work, summarizing key insights and discussing future research directions for enhancing RRM in the context of evolving 6G networks.

## 2. Capabilities of IMT-2030

6G extends beyond the advancements of 5G. As the latter one emphasizes enhanced mobile broadband (eMBB), massive machine type communication (mMTC), and ultra-reliable low-latency communications (uRLLC), 6G points to an intelligent infrastructure with enhanced versions of the current applications and new capabilities [5,27,28,29]. Potential 6G service categories include sensory and cognitive services, global connectivity via integrated TN-NTN, autonomous systems and smart cities enabled by ISAC, high-precision positioning, immersive extended reality, and edge-based AI processing.

In the context of RRM, resource allocation and scheduling are closely linked to service categories, performance indicators, multiple access schemes, and QoS metrics. Table 1 indicates the examined studies that considered service categories in their research. Notably, even when investigating RRM in the context of 6G networks, diverse studies relied on service categories defined for 5G. This reliance is primarily due to the absence of formally defined service categories for IMT-2030 networks. The delay in defining these categories for 6G has been influenced by several factors, including disruptions caused by the COVID-19 pandemic. This has led to a slower pace in 6G research and development. Consequently, current RRM research often extrapolates 5G service categories to predict and address the needs of future networks.

As 6G evolves toward its anticipated deployment around 2030, the service categories are expected to be refined in response to ongoing research and emerging societal and industrial demands, supporting an increasingly diverse set of applications. It is worth mentioning that future service categories will necessitate novel RRM strategies to optimize resource allocation and scheduling, aiming for efficient and reliable network performance [30,31,32].

**Table 1:** Service categories considered in related works.

Service	Generation	Related Works
mMTC/IoT	5G	[33,34,35,36,37,38,39,40]
eMBB	5G	[41,42,43,44,45,46]
URLLC	5G	[41,42,43,44,45,46,47,48,49]
Massive URLLC	6G	[50]
Further eMBB	6G	[51]
Enhanced URLLC	6G	[51]
Long Distance and High-Mobility Communications	6G	[51]

**Figure 2:**
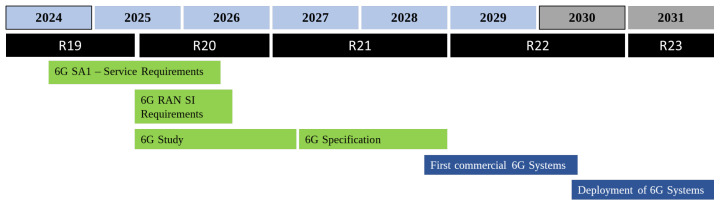
3GPP timeline for 6G development [52,54,55,56,57].

3GPP, known for creating the specifications for third, fourth and fifth generations of cellular networks (3G, 4G, and 5G), is currently dealing with the 6G development. Figure 2 presents the established timeline for this multi-year task. In mid-2024, 3GPP officially committed to developing 6G. The period from 2024 to 2026 is dedicated to defining service requirements for 6G, with emphasis on user needs, applications, and technical capabilities. By mid-2025, a study period began to explore technological options for 6G, concluding in 2027 with a comprehensive report. The first set of 6G technical specifications is expected by the end of 2028, and commercial 6G systems could be introduced by 2030, followed by a gradual global rollout [52,53,54,55,56,57].

**Figure 3:**
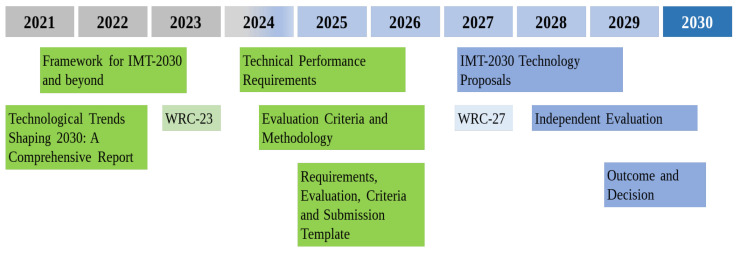
ITU standardization timeline for 6G (IMT-2030) [52,54,55,56,57].

Additionally, ITU plays a crucial role in standardization by defining the criteria for a system to be classified as an IMT technology. IMT classification is important as it grants access to a broad spectrum of frequency bands globally or regionally recognized as IMT bands, which is a significant step towards creating a global commercial ecosystem for 6G [52,56]. ITU has designated 6G as IMT-2030, organizing various tasks, some of which have already been completed, while others are just beginning. These tasks encompass an approach to 6G development, addressing multiple facets such as spectrum management, technical performance requirements, and evaluation methodologies. This systematic progression ensures that 6G technology evolves in alignment with global standards, facilitating its adoption and implementation worldwide [52]. Figure 3 illustrates the ITU standardization timeline for 6G. One can note this process starts with a report on technological trends and moves on to technical specifications toward technology proposals, independent evaluation, outcome and decision up to 2030 [52,54,55,56,57].

### 2.1. 6G Key Performance Indicators

Each generation of cellular communications introduces updated KPIs, and 6G is no exception. Early projections for 6G KPIs indicate both conservative and ambitious targets, all suggesting substantial improvements over 5G. Engineers working on 6G face not only technological but also ethical challenges, including mitigating social biases that may result in unequal access to wireless services. Overall, 6G aims to improve quality of life [32].

IMT-2030 is expected to significantly extend the capabilities introduced in IMT-2020, supporting broader and more advanced usage scenarios. These capability values are currently considered research targets and will be refined based on factors such as frequency range, bandwidth, and deployment conditions. Not all performance targets are expected to be achieved simultaneously within a single scenario [5,32,58].

In the early stages of 6G research, a diverse group of researchers and visionaries is actively defining use cases and system requirements. Comparative analyses (e.g., spider diagrams) highlight differences between 5G and 6G in metrics such as latency, throughput, and localization accuracy. While some KPIs represent incremental improvements over 5G, others emerge from novel functionalities envisioned for 6G [32,58]. It is worth mentioning that 6G KPIs represent idealized targets and are subject to future revision. Table 2 presents a summary of KPIs for 6G discussed in the literature, along with a comparative analysis against those from 5G.

Table 2 shows that all 5G performance indicators are expected to be preserved and further improved in 6G networks, reflecting the evolution required to meet increasing service demands while improving QoS and user experience. The ambitious targets will increase RRM complexity, requiring adaptive and dynamic algorithms for efficient resource allocation and scheduling.

Notably, energy efficiency, defined as the amount of information transmitted or received per unit of energy consumption, not explicitly defined for 5G, is now widely recognized as a critical metric for 6G. The accuracy of positioning indicators becomes significantly more relevant, especially with the anticipated ISAC in IMT-2030 networks. Indicators such as peak data rate, user experienced data rate, and maximum bandwidth are magnified 50-fold, 10-fold and 100-fold, respectively. Frequency range is expected to reach the visible light spectrum, receivers should deal with power levels below −130 dBm, mobility and peak spectrum efficiency are targeted to increase by twice, whereas latency should decrease 10-fold for real-time applications. Significant enhancements are also predicted regarding connection density for supporting billions of IoT devices and reliability toward mission-critical applications [5,29,32,58,59].

Additionally, several new capabilities are envisioned as key performance indicators for 6G networks according to ITU, though they have not yet been precisely quantified. These include [5]:

- Security and Resilience: Ensuring robust protection against cyber threats and maintaining network stability under various conditions to support mission-critical applications.
- Coverage: Extending network reach to remote and underserved areas, including deep-sea and space communications, to ensure global connectivity.
- Sensing Capabilities: Enhancing the network’s ability to sense and respond to environmental changes, which can support applications such as smart cities, autonomous driving, and industrial automation.
- AI-Related Capabilities: Integrating advanced artificial intelligence to optimize network operations, improve decision-making, and enable new services through predictive analytics and automation.
- Sustainability: Focusing on energy efficiency and minimizing the environmental impact of network operations, aligning with global sustainability goals.
- Interoperability: Ensuring seamless interaction between different technologies and standards, facilitating the integration of diverse communication systems and devices.

The capabilities discussed collectively represent a forward-looking vision for 6G, which requires advanced RRM techniques to meet the stringent requirements. RRM will prioritize low-latency traffic, optimize network slicing, implement rapid resource scheduling algorithms, and ensure high reliability through redundant paths, robust error correction, and swift failure recovery mechanisms. From the IoT perspective, RRM will handle vast numbers of simultaneous connections efficiently, a task that involves effective spectrum management, dynamic resource allocation, and advanced interference management techniques. Regarding energy aspects, RRM will optimize energy consumption using sleep modes, energy-efficient transmission schemes, and energy-aware scheduling. RRM must also incorporate secure communication protocols, encryption and privacy-preserving techniques for security and privacy while maintaining performance efficiency, as well as integrate AI and ML for automated decision-making and network optimization. It is worth highlighting that spectrum and energy are two valuable radio resources that are scarce and expensive; in such a way, advanced RRM techniques will play an important role in optimizing such resources and enabling sustainability and cost-effectiveness.

### 2.2. Energy: Balancing Innovation and Sustainability

Wireless communication systems, including mobile networks, are a relevant way for our increasingly connected world. However, their operation comes at an environmental cost. These systems consume significant resources, primarily electricity and raw materials, throughout their lifecycle. With the rapid growth of data traffic, the energy demands are expected to increase substantially [32]. For instance, data transmission networks consumed 310 TWh in 2022, representing approximately 1.3% of global electricity use, underscoring their environmental impact [67].

The introduction of 6G networks presents both opportunities and challenges. While 6G promises faster speeds, lower latency, and enhanced network capacity, it also carries the potential for increased energy consumption unless significant focus is placed on energy efficiency. Research by the Global System for Mobile Communications Association (GSMA) Intelligence indicates that RAN, responsible for connecting devices to the core network, accounts for a staggering 73% of the energy used by mobile network operators. Additionally, the growing trend of network virtualization, cloud computing, and edge computing contributes to a continuous rise in energy demands from data centers [32].

To navigate this challenge, mobile network operators are actively exploring and implementing various strategies. Transitioning to renewable energy sources, such as solar and wind power, is an important step towards reducing reliance on fossil fuels and achieving net-zero emissions. Furthermore, research and development efforts are directed towards developing energy-efficient network architectures, protocols, and hardware components. Optimizing network traffic management and employing innovative techniques for network resource allocation can also contribute significantly to reducing energy consumption.

Sustainability is central to IMT-2030, which targets reduced power consumption, lower greenhouse gas emissions, and improved lifecycle management through circular economy principles (reuse, repair, recycling), while aligning with the Paris Agreement goals. IMT-2030 also promotes energy-efficient technologies (e.g., energy harvesting, backscatter communication, on-demand access) and supports cross-industry decarbonization via digital transformation. Achieving these goals requires standardized regulations, reduced raw material usage, life cycle assessment tools, and sustainable information and communication technology (ICT) practices [5,68].

In the context of 6G networks, wireless energy harvesting emerges as a pivotal technology for sustainable operation. It encompasses various scenarios, including dedicated radiofrequency (RF) harvesting, interference-aware harvesting, and ambient sunlight harvesting. However, the energy harvested from these sources varies significantly, necessitating intelligent power control for energy-harvesting devices [2,29].

As the number of connected devices and non-terrestrial network nodes grows, energy harvesting becomes relevant for sustainable wireless communication system evolution. Natural energy sources like solar, wind, and thermal are eco-friendly but suffer from unpredictability and periodicity, making reliable network service challenging. RF energy harvesting offers a solution by converting radio signals into valuable electromagnetic-based power, potentially transforming interference into a beneficial energy source [2,29].

Ambient backscatter communication (AmBC) is a promising technology due to its low cost, ease of deployment, and high energy efficiency. AmBC utilizes ambient RF signals from non-dedicated sources to power communication devices, enabling ultra-low-power communication and energy harvesting for IoT devices. These devices can transmit information by reflecting and modulating incident RF signals without requiring power-hungry RF transmitters. This method supports green IoT applications by combining wireless information and power transfer. It is worth mentioning that AmBC systems are vulnerable to wireless attacks like eavesdropping, spoofing, and jamming due to the broadcast nature of backscatter channels and the limited computational and energy resources, which complicate the application of complex cryptographic mechanisms for security [2].

Among the resources managed by RRM in the reviewed papers, power consumption has received the most research attention. This focus on power has led to the emergence of power control techniques as the dominant method for optimizing resource usage within RRM frameworks.

The remainder of this subsection focuses on the critical role of energy management within the framework of 6G RRM. Various works examined for this survey have explored power control, energy consumption reduction, and energy efficiency in 6G networks. A central aim of summarizing these studies is to provide insights into the diverse range of technologies, methods, and approaches that have been implemented to optimize energy use and promote sustainability.

Technologies like RIS, mMIMO, and advanced beamforming techniques have proven to be instrumental in enhancing energy efficiency. For instance, RIS enables dynamic adjustment of propagation environments, minimizing energy loss and reducing power requirements across diverse network scenarios. Similarly, mMIMO leverages spatial multiplexing to improve spectral and energy efficiency, reducing the need for excessive transmission power while supporting a higher number of devices.

From a methodological perspective, techniques such as ISAC, the use of unmanned aerial vehicles (UAVs) and high-altitude platforms (HAPs), along with AI-driven resource allocation algorithms, have demonstrated significant potential in reducing energy consumption. ISAC, for example, allows joint optimization of sensing and communication functions, leading to reduced overhead and more efficient power usage. UAVs and HAPs, in turn, offer flexible and energy-efficient solutions for extending network coverage in rural or hard-to-reach areas while minimizing the energy footprint compared to traditional terrestrial networks.

Regarding network architecture, the cell-free paradigm and the integration of terrestrial and non-terrestrial networks have shown great promise in improving sustainability outcomes. In CF networks, user-centric distributed APs reduce the reliance on high-power centralized base stations (BSs), thus lowering the overall energy consumption. Meanwhile, TN-NTN integration enables seamless communication between terrestrial and satellite components, optimizing power control through intelligent load balancing and reducing the energy costs associated with handovers and signal propagation in large-scale environments.

Network slicing and customized algorithms are also pivotal approaches contributing to energy savings. Slicing allows for tailored resource allocation based on service requirements (e.g., IoT, URLLC, and eMBB), ensuring that each slice operates with the minimum energy needed to meet its QoS targets. Meanwhile, custom algorithms, often AI- or ML-based, optimize the trade-offs between performance and energy usage, making real-time adjustments to power control, beamforming, and resource allocation.

Recent studies demonstrate diverse optimization approaches: (i) multi-agent deep reinforcement learning (MADDPG) for joint resource allocation and power control [35]; (ii) optimized power allocation in NOMA systems, showing limited gains from joint transmission under certain conditions [69]; (iii) symbiotic radio systems with optimized scheduling for energy-efficient IoT communication [37]; (iv) UAV-assisted cognitive radio networks with energy harvesting and convex optimization for improved energy efficiency [70]; and (v) virtual slicing with fog computing achieving ~30% energy efficiency gains [71]. Additional contributions include joint RAN-core resource allocation, reducing energy consumption by 34% and cost by 24% [44]; semantic communication with probability graph compression to minimize communication–computation energy trade-offs [72]; dynamic resource allocation and puncturing strategy (DRAPS) for efficient coexistence of eMBB and URLLC services [46]; and RIS-assisted Mobile Edge Computing (MEC) systems enabling low-power computation offloading via joint optimization of radio, computing, and environment parameters [73]. Overall, energy-efficient design in 6G requires integrated advancements in architecture, algorithms, and enabling technologies to balance performance growth with sustainability objectives.

### 2.3. Frequency Spectrum Utilization in 6G Networks

The emergence of 6G networks promises a substantial leap in spectral efficiency (SE), potentially exceeding current 5G capabilities by a factor of ten. This improvement, as summarized in Table 2, could enable significantly higher data rates within the same amount of radio spectrum. However, estimates vary. While some projections are optimistic, ITU adopts a more conservative perspective, anticipating gains between 1.5 and 3 times, reflecting practical deployment constraints and technological challenges [5].

To achieve the ambitious SE goals of 6G, researchers are exploring advanced techniques, including mMIMO, AI-driven resource allocation, and intelligent reflecting surfaces, which improve signal transmission and reception capabilities [74]. Additionally, NOMA enables multiple users to share the same spectrum more efficiently [75,76], and the cell-free mMIMO paradigm eliminates conventional cell boundaries, enabling cooperative transmission and improved coverage. Other innovations include multi-carrier (MC) waveforms, orbital angular momentum, and millimeter-wave (mmWave) and THz spectrum utilization, all contributing to increased system capacity [74].

Although network densification improved capacity in 4G/5G, it increases cost and interference. Cell-free architectures bring advantages by enabling cooperative transmission without cell boundaries. Similarly, cloud radio access network (C-RAN) enhances efficiency through base station cooperation and densification gains [3,77].

The expansion to mmWave and sub-THz bands (100–300 GHz) enables higher data rates but limited coverage, requiring advanced multi-connectivity solutions to aggregate resources across different frequency bands while maintaining reliability and flexibility [3]. ITU emphasizes that no single frequency band can meet all IMT-2030 requirements. Instead, multi-band operation, from sub-GHz to above 100 GHz, is required: low bands ensure coverage, mid bands balance coverage and capacity, and high bands provide ultra-high throughput in short areas [5]. It is well-known that THz faces severe path and absorption losses; however, advances in ultra-mMIMO and beamforming help mitigate these challenges.

The transition to 6G requires a shift from static to dynamic spectrum management. Dynamic spectrum access (DSA) allows frequency bands to be shared across various operational contexts—commercial, governmental, licensed, unlicensed, and across different spectrum rights holders—along dimensions such as frequency, time, space, and direction. This flexible approach, supported by AI and blockchain, aims to enhance spectrum efficiency and support innovative wireless services [3,30].

Effective regulation remains important as the telecommunications sector evolves, particularly in the context of 6G, RRM, and spectrum usage. With a focus on efficient spectrum access, electromagnetic field compatibility, and fair competition with platform and cloud operators, regulation will play a significant role by 2030. Future networks will likely exploit HetNet and integrate terrestrial and non-terrestrial systems, requiring refined policies for dense, cell-free deployments [3,30].

Cell-free mMIMO systems have been evaluated for SE in [36], demonstrating significant improvement. In scenarios where each user and access point (AP) is equipped with a single antenna, studies show up to a five-fold increase in spectral efficiency for 95% of users using the cell-free approach compared to traditional cellular architectures. Further improvements in SE can be achieved by equipping users and APs with multiple antennas [78,79].

Considering 6G RRM, ref. [36] investigates a dynamic CF framework in a 6G-enabled IoT network to handle the increasing number of connected devices and the high volume of data traffic, which requires efficient frequency spectrum utilization. In this framework, many APs are distributed over a geographic area, serving multiple user nodes. The paper introduces a pilot-based AP Selection (PBAS) algorithm, which improves spectral efficiency by selecting only a subset of APs for each user node, rather than engaging all available APs. This approach optimizes the allocation of frequency spectrum resources while maintaining seamless communication and supporting massive connectivity.

Further analysis in [36] evaluates different receive-combiners for the proposed communication model using the PBAS algorithm. Minimum mean square error receive-combining performs comparably to partial minimum mean square error-combining, achieving a spectral efficiency of around 4.4 bits/s/Hz for cell-edge user nodes and 13.0 bits/s/Hz for cell-center user nodes. Maximal ratio-combining provides the lowest performance, with a minimum spectral efficiency of around 0.31 bits/s/Hz and a maximum of 10.5 bits/s/Hz. Partial regularized zero forcing combining outperforms maximal ratio-combining, offering a spectral efficiency of around 12.8 bits/s/Hz for cell-center users.

In summary, ref. [36] highlights the potential of CF mMIMO with dynamic AP selection for enhancing spectral efficiency in 6G IoT networks. The proposed approach offers significant gains, especially for users located at the cell edges. Additionally, the study emphasizes the importance of selecting appropriate receive-combining techniques to further optimize system performance.

The paper [74] also investigated RRM, and the authors believe that innovations in multiple access and modulation techniques, particularly focusing on filter-bank multicarrier (FBMC), have enabled significant advancements in the spectral efficiency of 6G networks. The work emphasizes the critical role of MC modulation in achieving high data rates and spectrum efficiency, proposing FBMC as a strong candidate for 6G. The authors compared FBMC with other modulation techniques based on factors such as spectrum efficiency, advantages, drawbacks, and MIMO flexibility. They identified FBMC as an alternative waveform to orthogonal frequency division multiplexing (OFDM) and a potential 6G modulation technique due to its unique features. These features include its pulse shaping filter, which results in low out-of-band emissions (OOBEs), minimal intersymbol interference (ISI), and cyclic prefix (CP)-free transmission, all of which enhance spectral efficiency and data rate. Additionally, FBMC is highly suitable for the THz band, a candidate for 6G frequencies, due to its vast bandwidth and immunity to RF interference.

NOMA is another key technology, enabling simultaneous multi-user transmission over shared resources and achieving higher spectral efficiency than orthogonal multiple access (OMA), particularly in dense and heterogeneous networks [75,76,80]. The combination of NOMA and mMIMO further enhances performance through spatial multiplexing, interference mitigation, and improved fairness, making it essential for beyond 5G (B5G)/6G systems. In integrated TN–NTN, spectrum sharing improves utilization but introduces interference challenges, requiring advanced resource management and mitigation strategies. Approaches include cooperation mechanisms, cognitive spectrum use, and NOMA-based integration [81]. Finally, network slicing, MEC, and NOMA jointly enhance spectral efficiency. Studies show that NOMA outperforms OMA, particularly as the number of users increases, making it a strong candidate for future 6G systems [48]. Notably, 3GPP considered the use of NOMA in emerging wireless communication systems in its Release 15.

### 2.4. Advanced Multiple Access Techniques for 6G Networks

The transition to 6G demands the development of advanced and hybrid multiple access schemes [82]. Table 3 provides a representative sample of the primary multiple access techniques examined in various research works on RRM. While not exhaustive, it highlights the key methods considered. The NOMA technique emerged as the most frequently considered method in the surveyed studies on 6G RRM, featuring in 49% of the works. Orthogonal frequency division multiple access (OFDMA) was also extensively analyzed, appearing in 39% of the papers. The inclusion of multiple access techniques in 75% of the works suggests that hybrid solutions, combining NOMA with other multiple access methods such as OFDMA, sparse code multiple access (SCMA), delta-orthogonal multiple access (D-OMA), or orthogonal time frequency space (OTFS), might be a promising direction for future research.

As 6G aims to support diverse applications with vastly different requirements, OFDMA remains a critical technology due to the flexibility in resource allocation. NOMA, in particular, has garnered significant attention due to its potential to enhance both spectral and energy efficiency. By allowing multiple users to share the same frequency resources, NOMA improves overall system throughput while mitigating the limitations posed by traditional orthogonal schemes. The prominence of NOMA in nearly half of the surveyed works indicates its strong appeal for addressing some of the most pressing challenges in 6G. With the promise of massive connectivity, NOMA enables the simultaneous servicing of numerous devices, ranging from IoT sensors to high-bandwidth applications, without requiring additional bandwidth or spectrum. This capability is desirable for supporting the diverse traffic profiles anticipated in 6G. The high adoption of NOMA also highlights its importance in achieving energy efficiency and spectrum utilization. By superimposing signals of different users at varying power levels, NOMA can effectively reduce transmission power while maintaining adequate service quality, thereby contributing to sustainability efforts in energy-constrained environments. This aspect aligns with the broader industry trend of designing networks that are not only high-performing but also energy-conscious.

NOMA can be broadly categorized into power-domain NOMA and code-domain NOMA. Power-domain NOMA differentiates users by assigning them different power levels, relying on successive interference cancellation at the receiver to separate the superimposed signals. This method is particularly effective for users with varying channel conditions. On the other hand, code-domain NOMA employs unique spreading codes for each user, enabling simultaneous transmission and separation based on these codes. Both types of NOMA aim to maximize spectral efficiency and support massive connectivity, which are critical for the proliferation of IoT devices and high-demand applications in B5G/6G networks [99,100].

In the specific context of 6G RRM, NOMA plays a pivotal role in optimizing the allocation of limited radio resources such as spectrum and transmission power. By allowing multiple users to share the same resources, NOMA enhances the overall utilization of the electromagnetic spectrum and improves network throughput. Effective RRM in 6G involves sophisticated algorithms to manage power levels, minimize interference, and ensure QoS for diverse applications. Researchers are exploring various NOMA-based strategies to address these challenges, such as integrating NOMA with multi-carrier waveforms and developing advanced heuristics for power allocation [100,101,102].

In [75], the authors introduced a new framework called the ensemble metaheuristics optimization method to maximize total throughput in MC cell-less NOMA scenarios by efficiently allocating transmission power levels in base stations. Such a framework involved three stages: identifying the most suitable BS for each user device, determining the appropriate sub-carrier allocation, and using an ensemble algorithm that employs four metaheuristics in parallel to allocate transmission power levels. Empirical analysis with real-world data validated the framework’s viability, demonstrating high throughput rates and low outage probabilities. Also targeting enhancement of the radio throughput, the authors in [40] combined NOMA, orthogonal multicarrier and multihoming technology. An iterative joint device assignment and power allocation algorithm was proposed, demonstrating improved performance and connectivity.

Article [33] investigated cooperative resource allocation for a hybrid NOMA-OMA-based wireless powered multi-carrier IoT system. Two cooperative protocols were proposed: hybrid NOMA-FDMA and hybrid NOMA-TDMA. In the former, relays and IoT devices (IoTDs) operate on dedicated channels, whereas in the latter, they utilize all channels within assigned time slots. Consequently, these protocols improve coordination between energy transfer and information transmission, enhancing overall system efficiency.

In [35], the use of NOMA for dynamic resource management in integrated terrestrial-satellite networks was discussed. The research employs multi-agent reinforcement learning to optimize resource management, leveraging interference cancellation and superposition coding to cluster users into NOMA groups and prioritize those with better channel conditions, thereby minimizing interference and enhancing overall network performance. By exploiting a cognitive satellite-UAV network, the authors in [97] proposed a framework for on-demand coverage in remote areas. Multiple UAVs form a virtual antenna array using MIMO-NOMA, while a radio map containing large-scale channel state information (CSI) supports interference mitigation and coordinated transmission. A joint power allocation problem is formulated to maximize the minimum user rate. Simulation results confirmed performance gains despite coarse CSI granularity, demonstrating a cost-effective solution for 6G coverage and resource allocation.

Paper [48] discussed the integration of NOMA within a cell-free RAN for 6G, highlighting gains in spectral and energy efficiency as well as fairness. The study combines power-domain and spatial-domain techniques to mitigate interference among users sharing the same resource blocks (RBs). It details the process of allocating virtual communication resources to slices and further distributing RBs to NOMA clusters within each slice. Users within these clusters are organized based on their channel gains, with stronger users decoding messages first using successive interference cancellation.

In [98], a federated quantum neural network (FT-QNN) with quantum teleportation was introduced for NOMA power allocation optimization. The framework integrates edge and cloud quantum neural networks, allowing direct quantum-state transmission without measurement. The target was sum-rate maximization while reducing computational complexity. Furthermore, FT-QNN addresses scalability issues in B5G/6G networks with many antennas and devices. By leveraging quantum learning, it achieves faster convergence and lower complexity compared to classical methods, demonstrating strong potential for advanced wireless resource management. Complementary, paper [50] exploited the development of a novel learning framework for optimizing grant-free (GF) NOMA systems in the context of massive uRLLC. It introduces multiple configured-grants (MCG), enabling user equipment (UE) to transmit immediately upon data arrival, thus reducing latency and congestion. The system models each grant using parameters like contention–transmission units, slot timing, and repetitions, formulating an optimization problem for latency and reliability. Next, a cooperative multi-agent double deep Q-network algorithm was developed to solve the configuration problem. Simulation results showed that MCG-GF-NOMA serves approximately four times more UEs and halves latency compared to single-configured grant-GF-NOMA under high traffic.

## 3. Network Geographical Arrangement

The success of the RRM strategies depends on the 6G network geographical arrangement. This section delves into the CF and integrated TN-NTN architectures and how they impact the RRM functionalities. Table 4 presents a summary of the geographical arrangements for 6G systems based on the papers about RRM investigated for this work.

One can note that the majority of the investigated articles focused on 6G networks with terrestrial cellular infrastructure in 2D. Within this context, most of these studies considered heterogeneous networks. It can be observed that while the networks investigated may be heterogeneous and envisioned for 6G, they are primarily terrestrial and cellular in nature. This highlights research demands and opportunities on 6G geographical arrangement.

### 3.1. Cell-Free Network

**Figure 4:**
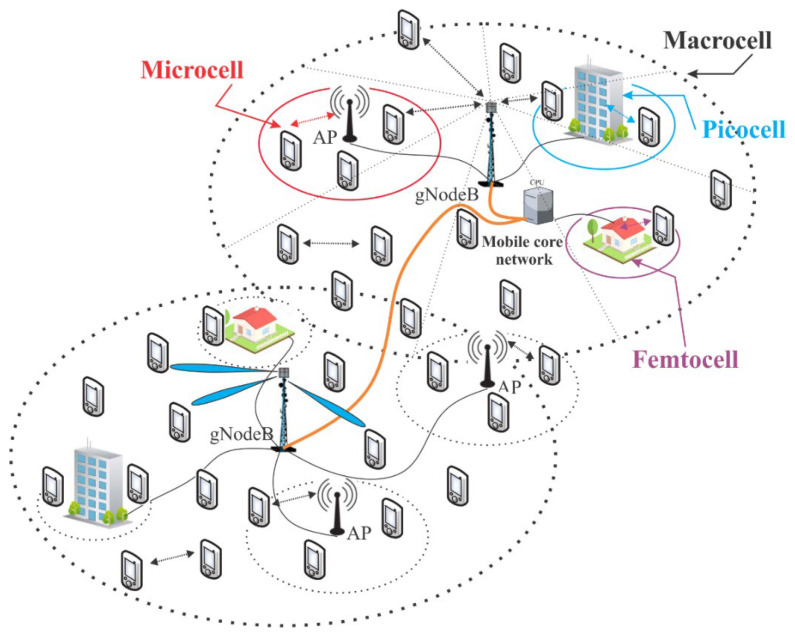
Typical heterogeneous terrestrial 5G network architecture.

Figure 4 illustrates a typical heterogeneous terrestrial 5G network architecture comprising a mix of cell sizes, namely macrocell, microcell, picocell, and femtocell. One can note that the 5G planning considers macrocells overlay and smaller cells underlay, as well as a high density of network nodes with varying coverage radii and power levels, toward user equipment experience continuity. In such cellular network architecture, the infrastructure of a specific service provider allocates and schedules radio resources based solely on local knowledge of the radio environment to meet the demand and requirements of users within its coverage area. This creates a competitive environment where internal competition for resources can lead to suboptimal allocation [85,120].

6G systems promise to revolutionize the traditional cell concept. To achieve ubiquitous coverage, accommodate the growing number of connections, and meet the demands for higher data rates and reduced latency, two key alternatives are proposed: (1) replacing the cell concept with a CF or cell-less (CL) architecture, and (2) integrating terrestrial and non-terrestrial communication infrastructure [36,43,48,51,75,81,85,86,87,121,122,123,124].

**Figure 5:**
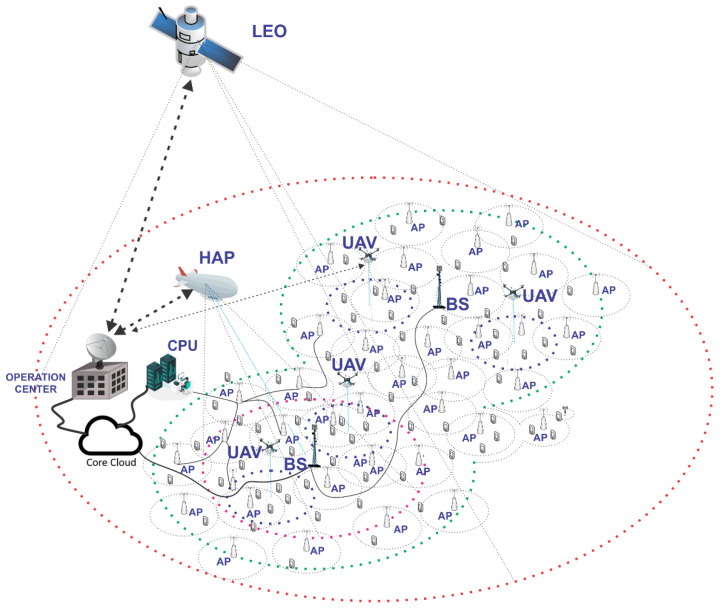
Future 6G network architecture, illustrating the evolution toward a 6G 3D-heterogeneous network (3D HetNet).

Figure 5 illustrates a future 6G network architecture including both alternatives. This ecosystem envisions the concurrent operation of 5G and 6G systems. The 6G network itself comprises a dense network of APs. Additionally, NTN uses satellite connections at various altitudes (low Earth orbit—LEO, medium Earth orbit—MEO, geostationary Earth orbit—GEO) and high-altitude platforms (HAPs) to complement TN. Unmanned aerial vehicles can also be integrated to provide localized coverage, whereas satellites and HAPs offer broad geographic coverage, particularly beneficial in remote areas. The researchers recognize that cell-free and cell-less are distinct concepts. However, both utilize the principles of mMIMO and beamforming to disrupt traditional cellular networks with defined borders. Instead of the competitive model for resource allocation and scheduling, they adopt a cooperative scheme that leverages intense spatial diversity. This approach enhances network performance by improving coverage and capacity through collaborative use of network resources. Moreover, Figure 5 illustrates the evolution toward a 6G 3D-heterogeneous network (3D HetNet), an ecosystem that transcends traditional 2D planar connectivity by integrating dense terrestrial 5G/6G access points with a multi-layered NTN. Central to this vision is the transition from rigid, competitive cellular borders to a user-centric, cell-free paradigm. By leveraging Massive MIMO and advanced beamforming, the network replaces traditional resource scheduling with a cooperative scheme that utilizes intense spatial diversity. This collaborative approach allows multiple distributed access points to serve a single user simultaneously, effectively eliminating cell-edge interference and providing the seamless connectivity required to meet 6G’s ambitious targets.

In conventional cell-free mMIMO systems, all APs are connected to a central processing unit (CPU), which coordinates the APs to function as a unified mMIMO network without cell boundaries, enabling coherent transmission and reception to serve all users seamlessly. Specifically, the CPU enables all APs to serve all users simultaneously using the same time-frequency resources by employing spatial multiplexing techniques [122,123,124]. However, conventional CF mMIMO systems face scalability challenges and issues related to fronthaul signaling and high computational complexity, rendering them impractical for large-scale networks [81,84]. To address these issues, a more practical approach called user-centric CF mMIMO has been proposed. The major distinction is that user-centric CF mMIMO explicitly defines a serving cluster for each user, thereby limiting the number of serving transmitters. In contrast, conventional CF mMIMO theoretically assumes that each user can be jointly served by all transmitters in the network [123,124,125].

Cell-less networks take the concept of CF networks a step further by completely eliminating the notion of cells. Instead of relying on a fixed infrastructure to define network coverage areas, CL networks rely heavily on intelligent surfaces, device-to-device communication, and extreme densification of network nodes. In this architecture, the network adapts to user locations and varying network conditions. Users connect directly to any available access point in the network, forming dynamic user-clusters. This highly adaptive and flexible approach ensures seamless connectivity and optimizes network performance in real time [75,85].

Both network models, CF and CL, are envisioned to meet the stringent performance indicators of 6G, including enhanced spectral and energy efficiencies, high data rates, and ultra-low latency. Additionally, CF and CL networks will significantly enhance the reliability, flexibility, and scalability of 6G systems [121,124,125].

These networks employ a distributed topology and ultra-densification, providing unprecedented levels of macro-diversity gain. This results in more reliable communication links since multiple APs serve each user, thereby reducing the probability of signal blocking. Furthermore, with APs being closer to users, path loss and shadowing effects are minimized, leading to higher channel gains and overall improved communication performance [123,125,126].

Cell-free mMIMO networks are envisioned as a cornerstone technology for future 6G deployments. However, significant challenges need to be addressed before widespread adoption. The challenges include:

- Synchronization: CF mMIMO relies on precise coordination and uses a large number of distributed APs working together. These APs need to be tightly synchronized and transmit signals with minimal distortion to ensure efficient beamforming, accurate signal combining at the user equipment [123,126].
- Hardware Impairment: Hardware imperfections disrupt coordination in CF mMIMO networks. Real-world hardware components in the APs have unavoidable shortcomings that can cause phase noise, gain and phase imbalances, quantization errors, and other issues. Additionally, the large number of multi-antenna APs in CF mMIMO systems leads to substantial energy consumption and elevated hardware costs [122,126].
- Estimation of Channel State Information: Unlike traditional cellular networks with dedicated base stations, CF mMIMO relies on multiple distributed APs serving users simultaneously. Accurate CSI is essential for beamforming, precoding, and interference management. The major challenges in estimating CSI for CF mMIMO include fast-changing channels, pilot contamination, and high computational complexity [123,125].
- Fronthaul Bottleneck: In CF mMIMO networks, a critical challenge lies in the ever-increasing volume of data exchanged between APs and the CPU for real-time coordination and signal processing. As the network scales with more APs and users, the fronthaul data traffic grows exponentially, demanding high-capacity fronthaul links for maintaining the benefits of CF mMIMO in large-scale deployments [122,126,127].
- Energy Efficiency: The substantial deployment of geographically distributed APs in CF mMIMO networks poses a significant energy efficiency challenge. As more APs engage in active data transmission and processing, the overall network power consumption escalates considerably. This increase in energy usage can result in higher operational costs and a greater environmental impact. To enhance energy efficiency, it is required to employ various strategies, including network densification optimization, power control and scheduling algorithms, and the development of energy-efficient hardware.
- High-Mobility Scenarios: Addressing high mobility in CF mMIMO networks requires advanced channel prediction, efficient handover mechanisms, robust synchronization, and adaptive antenna array designs. The communication channel between the user and APs changes rapidly due to factors such as Doppler frequency shift and time-varying channels (e.g., fast fading), resulting in frequent handovers and spatial resolution challenges. Specifically, the channel coherence length decreases, making it difficult to distinguish different paths, and the system must adapt quickly to changes in the channel’s spatial characteristics. Additionally, antenna array design must account for beamforming accuracy to maintain effective communication.

The convergence of 6G and CF mMIMO presents numerous research opportunities in RRM, particularly in resource allocation and scheduling. The following are some of these opportunities:

- Intelligent Resource Allocation with User-Centric Optimization: User-centric allocation, which considers individual user needs and locations, can lead to improved network efficiency and enhanced user fairness. Hence, by developing algorithms that consider user location, channel conditions, and application requirements (e.g., low latency, high bandwidth or high rates) may be possible to dynamically allocate resources. Machine learning techniques can be used to analyze vast amounts of user data and network information for real-time optimization.
- Joint Optimization of Resource Allocation and Scheduling: Optimizing resource allocation and scheduling together can lead to significant performance gains. Scheduling determines which users receive resources at specific times, while allocation determines the type and amount of resources assigned. Therefore, developing joint optimization algorithms that consider both factors simultaneously can improve the performance. This could involve exploiting the distributed nature of CF networks, where multiple access points can cooperatively serve users, leading to more efficient resource utilization and improved throughput.
- Mobility Prediction and Beamforming Strategies: mobility prediction helps anticipate user movement and optimize resource allocation and scheduling before disruptions occur. Beamforming focuses radio signals on specific users, improving signal strength and reducing interference. Then, constructing advanced mobility prediction algorithms that leverage user data, network information, and context-awareness (e.g., traffic patterns) to improve accuracy is an important issue. Integrating mobility prediction with beamforming strategies that can dynamically adjust signal directions based on predicted user movement is another opportunity.
- Exploiting Channel State Information Feedback: Accurate CSI feedback allows for informed resource allocation and scheduling decisions. This information describes the channel conditions between users and access points. So, designing efficient CSI feedback mechanisms that minimize overhead while providing reliable channel information, or project algorithms that can exploit this information for adaptive resource allocation and scheduling, optimizing network performance for different user needs, is important.
- Security and Privacy: Ensuring secure resource allocation and scheduling in CF networks is important to prevent unauthorized access and protect user privacy. Two important questions are: formulate secure user authentication and authorization procedures for resource allocation, and design privacy-preserving algorithms that allocate resources while minimizing user data exposure on the network.

### 3.2. Integration of Terrestrial and Non-Terrestrial Networks

The distinction of TN and NTN is based on the physical location of the network infrastructure relative to the Earth’s surface. TNs consist of above-ground or subterranean networks embracing radio base stations and/or APs that evolve with each generation of wireless technology [51,128,129], whereas NTNs comprise a variety of communication elements arranged in vertical layers, e.g., UAVs, HAPs and satellites, thus expanding coverage to remote areas or situations where terrestrial infrastructure is limited [54,115,130].

From Table 5, one can note that the majority of articles on 6G RRM focused on TN infrastructure, whereas a smaller percentage of studies considered 6G RRM within the scope of non-terrestrial communication modes, such as satellite or aerial networks. Few works evaluated 6G RRM with an integrated approach, combining both TN and NTN. This highlights a significant research gap in the holistic evaluation of 6G RRM across diverse network types. This distribution underscores the current focus and trends within 6G RRM research, emphasizing terrestrial infrastructures while indicating emerging interest and the necessity for more comprehensive studies that include non-terrestrial and integrated network environments.

The TN-NTN integration has already become a reality within 5G networks since the publication of 3GPP report 17 in 2022. However, the most significant gains from such integration are expected to materialize in the next generation of networks. It is worth mentioning that this integration considers existing 5G use cases while addressing their limitations and identifying new challenges specific to 6G, thus playing a pivotal role in enabling global coverage/connectivity and supporting a wide range of use cases [51,81,115,130,133,134,135,136,137].

Despite the potential benefits, integrated TN-NTN introduces several challenges and limitations, particularly from the perspective of researchers focusing on RRM, scheduling, and resource allocation. Firstly, the network’s complexity increases significantly due to the heterogeneous components and dynamic and random behavior of the environment. Researchers need to develop algorithms capable of managing a diverse network comprising terrestrial base stations, access points, communication devices, and satellites at various orbits, as well as aerial vehicles. Each component presents unique characteristics in terms of latency, capacity, coverage and mobility, which must be taken into account. Integrating mobile TN components with dynamic NTNs adds an additional layer of complexity. Consequently, RRM algorithms must adapt to rapidly changing network conditions to ensure seamless connectivity and optimal performance. It follows an overview of some additional challenges and limitations [115,133,134,135,136,138,139]:

- Latency and Handovers: A significant challenge is coordinating seamless handovers between terrestrial and non-terrestrial components, as maintaining low latency during these transitions is crucial for real-time communication.
- Interference Management: Interference management poses a challenge, with the need to ensure the coexistence of TNs and NTNs in shared spectrum without causing harmful interference between terrestrial and space-based systems.
- Resource Allocation and Scheduling: Optimal distribution of resources such as frequency, time, and power across both domains, balancing the diverse requirements of different services, from IoT to high-throughput applications, is required.
- Energy Efficiency: The NTN-TN is also a challenge, necessitating efficient management of energy consumption while balancing tracking accuracy for mobile NTNs with energy savings.
- Complex Orchestration: This is another hurdle, involving the harmonization of ground-based and aerial communication, which is complicated by regulatory considerations, licensing, and standardization issues.
- Edge Computing Integration: Integrating edge computing with NTNs to leverage edge processing and support low-latency applications is complex, as it requires seamless interaction between edge nodes and NTN components.

However, the ongoing research on RRM in 6G offers significant opportunities, as presented in the following:

- Advanced Handover Management: Develop efficient algorithms that minimize service disruptions during handovers between diverse network components. This involves predicting user movement, pre-configuring resources on target networks, and optimizing handover triggering mechanisms.
- Context-Aware Handover Decisions: Move beyond signal strength for handover decisions. Consider factors like user applications (latency vs. bandwidth needs), network congestion, and user mobility patterns.
- Latency-Aware Resource Allocation: Allocate resources with varying sizes (small blocks for low-latency, larger blocks for bulk data) based on network component capabilities and user needs.
- Dynamic Spectrum Sharing: Develop advanced techniques for efficient spectrum utilization across TNs and NTNs with minimal interference. Cognitive radio approaches and dynamic spectrum allocation are key areas of research.
- Interference Coordination: Design algorithms for inter-network coordination to mitigate interference between diverse network components operating on different bands.
- Advanced Scheduling Algorithms: Develop and implement intelligent scheduling algorithms that can efficiently allocate resources across TNs and NTNs based on real-time network conditions and user demands.
- Dynamic Allocation based on Demand: Create dynamic resource allocation strategies that can adapt to fluctuating network traffic patterns and user requirements.
- QoS-Aware Resource Management: Prioritize resources based on QoS requirements. Allocate resources with high reliability and low latency for mission-critical applications.
- Standardized Protocols and Interfaces: Develop and implement standardized protocols and open interfaces to ensure smooth interoperability between TN and NTN equipment from different vendors. This will enable seamless control and configuration of resources across the entire network.
- Open Research Platforms: Create open platforms for researchers and developers to test and refine new RRM algorithms and protocols for 6G networks with TN-NTN integration.
- Energy-Efficient Handover Strategies: Develop handover mechanisms that minimize energy consumption during network transitions.
- Adaptive Power Control: Implement algorithms for dynamic power control to optimize energy usage across the network, particularly for resource-constrained NTN components.
- Edge-Assisted Resource Allocation: Explore how edge computing can assist in real-time resource allocation decisions based on local network conditions and user needs.
- Computation Offloading: Investigate how to offload computational tasks to edge servers to reduce latency and improve network efficiency.
- Flexible Licensing Frameworks: Advocate for regulations that facilitate dynamic spectrum sharing and flexible licensing models to cater to diverse 6G network requirements.
- Harmonized Regulatory Policies: Promote international collaboration toward harmonized regulatory policies that enable seamless deployment and operation of global 6G networks with TN-NTN integration.

When TN-NTN integration is combined with HetNet architectures, a novel approach known as 3D HetNet emerges. It leverages network nodes at varying altitudes, including terrestrial, aerial and space-based components, creating a highly dynamic and flexible multi-layered network structure. The 3D architecture opens new opportunities for 6G RRM, particularly in terms of resource management across diverse physical layers and altitudes. It is worth noting that, even without integration, both TN and NTN operating independently can already be considered heterogeneous in nature, once TN incorporates diverse cell sizes while NTNs feature multi-altitude nodes like satellites, UAVs, and HAPs, thus inherently embodying the 3D concept. Despite similarities, TN-NTN integration and 3D HetNets have significant differences in scope, objectives, and implementation [128,129,133]:

- Scope: TN-NTN integration focuses on seamless communication between terrestrial and non-terrestrial nodes, ensuring coordination and minimizing coverage gaps. In contrast, 3D HetNets encompass a broader range of network nodes across different altitudes, emphasizing the creation of a truly 3D communication environment. This includes LEO satellites, HAPs, UAVs, and terrestrial stations.
- Objective: TN-NTN aims primarily at extending network coverage and enhancing the resilience of communications, particularly in remote areas, during disasters, or when terrestrial infrastructure is unavailable. 3D HetNets, on the other hand, aim to optimize overall network performance, employing a multi-layered approach to balance traffic, minimize latency, improve throughput, and manage spectrum efficiently. The 3D nature allows for strategic resource allocation and interference management across multiple dimensions.
- Implementation: TN-NTN integration involves sophisticated coordination protocols for seamless handovers between terrestrial and non-terrestrial nodes, ensuring that devices can transition smoothly between different network types. By contrast, 3D HetNets require advanced RRM techniques to allocate resources dynamically across various altitudes and layers. This includes handling interference, mobility management, and load balancing in complex 3D environments where different network nodes interact in real time.

## 4. Innovative RRM Approaches for 6G

**Figure 6:**
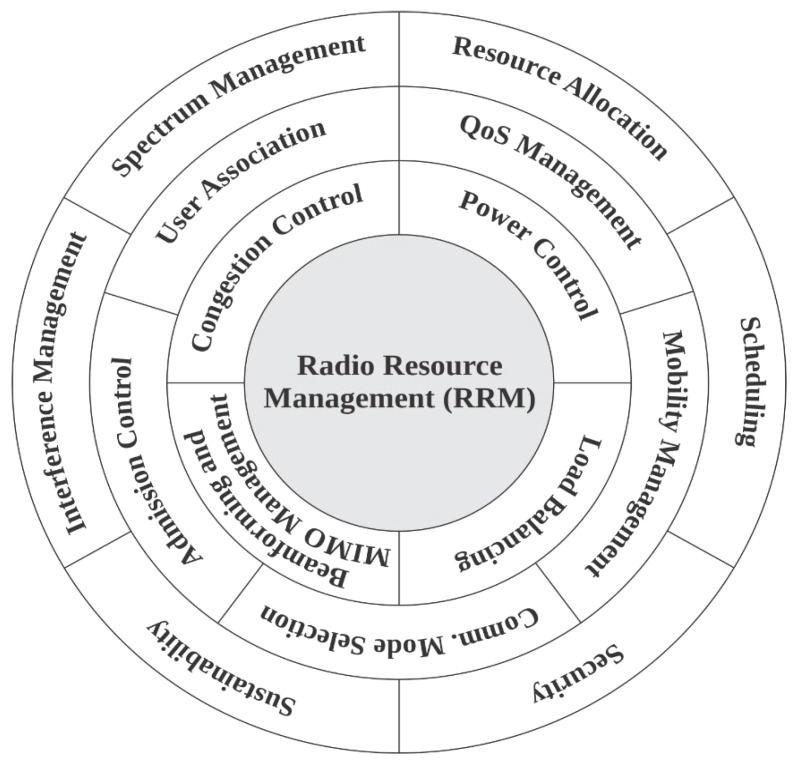
Anticipated functions of 6G radio resource management.

In a classical definition, RRM is the dynamic allocation, optimization, and control of wireless network resources, including spectrum, power, and antenna patterns, to maximize system performance, capacity, energy efficiency, and user experience while ensuring QoS and coverage [129,140,141,142,143,144,145]. Traditional RRM techniques can be applied to 6G networks to some extent, but they will require significant adaptation and enhancement to meet the unique demands and challenges of 6G. Figure 6 presents the anticipated functions of 6G RRM, which are briefly introduced hereafter.

### 4.1. Resource Allocation

The 6G resource allocation involves the dynamic and intelligent distribution of radio spectrum, time slots, power levels, and other resources among users and services. It leverages advanced technologies like AI and ML to maximize the network performance, QoS and QoE. The CF approach significantly enhances 6G resource allocation by utilizing distributed antenna systems and coordinated multi-point transmission. This improves user mobility, reduces handover frequency, and leads to more stable connections. When combined with mMIMO and beamforming technologies, CF networks achieve precise signal targeting and improved spectral efficiency, optimizing resource allocation and overall network performance, especially in dense urban environments.

ISAC and RIS also collectively enable more efficient and adaptive resource management, once ISAC provides precise user location tracking that allows networks to make informed decisions about resource distribution, and RIS further contributes to the improvement of signal quality and coverage. The integration of TN-NTN and 3D HetNets adds another layer of complexity and opportunity to 6G resource allocation. These technologies facilitate seamless connectivity across diverse environments, from urban areas to remote regions, and support a massive number of connections with high data traffic. By dynamically managing resources across these varied network layers, 6G RRM can efficiently meet the demands of a wide range of applications and services, ensuring robust and effective communication.

Table 6 presents a detailed list of the resources allocated in the RRM papers selected for this survey. Notably, frequency allocation was the most extensively investigated, featured in more than 70% of the publications. This is followed by power and energy allocation, which appeared in more than 50% of the papers analyzed. There has also been a notable increase in studies focusing on the allocation of computing resources, underscoring the growing demand for these resources and the necessity of deploying computing power closer to user equipment to accommodate the rising number of connections and service requirements.

The increasing focus on computing resources indicates a transformation in network functions. Networks have evolved from merely transporting voice signals to transporting, storing, and processing vast amounts of data. This trend is expected to intensify with the integration of AI in resource allocation, network function virtualization, network slicing, sensing technologies, and the adoption of service-based network architecture. These advancements underscore the necessity for innovative RRM strategies to handle the complexity and ensure efficient operation, ultimately enhancing the overall efficiency, reliability, and performance of modern wireless communication systems.

### 4.2. Scheduling

In the 6G RRM context, scheduling involves the strategic prioritization and timing of data transmissions to ensure fair and efficient use of network resources. This process is important for meeting QoS requirements across diverse applications and user demands. By employing advanced scheduling algorithms and techniques, such as round robin, proportional fair, and opportunistic scheduling, 6G systems can dynamically manage traffic loads, minimize latency, and enhance throughput. Utilizing AI and ML, these algorithms can adjust priorities in real time based on network conditions, user behavior, and service-level agreements. Additionally, efficient scheduling supports network slicing, enabling the accommodation of diverse and demanding applications, ultimately optimizing resource utilization and ensuring an optimal QoE for all users.

The CF approach significantly impacts scheduling by eliminating traditional cell boundaries and allowing distributed antenna systems to coordinate transmission. This approach reduces the complexity of handovers and improves user mobility, ensuring more stable connections and better resource utilization. In a CF network, scheduling algorithms can dynamically allocate resources based on real-time user locations and demands, enhancing spectral efficiency and network performance. This flexibility in resource management leads to more effective and efficient scheduling, especially in dense urban environments.

The integration of TN-NTN introduces new dimensions to scheduling by combining terrestrial and non-terrestrial components, such as satellites and high-altitude platforms. Such integration requires sophisticated scheduling strategies to manage the unique characteristics of each network layer. Scheduling in TN-NTN environments ensures seamless connectivity across diverse geographical areas, optimizing resource use and enhancing service delivery. These strategies enable the network to handle a massive number of connections and provide reliable service, even in remote or underserved regions.

ISAC, mMIMO, beamforming, and RIS also collectively contribute to more adaptive and efficient scheduling toward high QoS and QoE. ISAC allows for precise user location tracking, enabling more informed scheduling decisions that reduce interference and improve network performance. mMIMO and beamforming technologies enhance spatial resource management by directing signals where they are needed most, allowing for more efficient scheduling of transmissions. RIS dynamically alters the propagation environment to enhance signal quality and coverage, further optimizing scheduling strategies.

3D HetNets add another layer of complexity and opportunity to scheduling in 6G RRM. These architectures incorporate various types of base stations and user equipment across different layers and dimensions, requiring comprehensive scheduling strategies to manage the diverse characteristics and capabilities of each network element. Scheduling in 3D HetNets must balance the needs of different network layers to optimize overall performance, ensuring efficient use of resources and high service quality. By dynamically managing resources across these varied network layers, 6G scheduling can efficiently meet the demands of a wide range of applications and services, providing robust and effective communication.

Resource allocation and scheduling, coupled with interference mitigation, constitute the traditional core of RRM research. As shown in Table 7, the examined RRM literature reveals a consistent focus on resource allocation, with all studies incorporating this function within the 6G context. In contrast, scheduling received attention in ≈40% of the investigated works.

A significant gap in the current research is the lack of a comprehensive study on emerging 6G technologies. While individual components such as MIMO/mMIMO have been explored in about 40% of studies, there is a scarcity of research that integrates technologies like CF networks, ISAC, RIS, TN-NTN, and beamforming. For example, integrating CF networks with RIS and advanced beamforming could lead to more efficient use of the radio spectrum, improved coverage, and better signal quality. These combined effects might be greater than the sum of their individual contributions, leading to overall improvements in network performance, reliability, and efficiency that wouldn’t be possible by studying each technology separately. Furthermore, the majority of studies have isolated their analysis to either uplink (UL) or downlink (DL) channels, with less than 10% offering a comprehensive perspective on both. This limited scope hinders the development of cross-link optimization strategies for maximizing overall network performance. The limited focus on both uplink and downlink channels in the existing research highlights another area for improvement. Future research should aim to develop holistic approaches that consider the unique requirements and challenges of both UL and DL channels, along with the integration of multiple advanced technologies, to achieve a more robust and comprehensive 6G RRM framework.

In conclusion, while significant progress has been made in the field of RRM for 6G networks, there remains a considerable scope for further research. By embracing a more integrated and comprehensive approach, future studies can pave the way for the next generation of wireless communication, delivering unprecedented levels of connectivity, efficiency, and user satisfaction.

### 4.3. Interference Management

Reducing and managing interference between different users and devices is important for maintaining high-quality communication links in 6G networks. This becomes increasingly challenging due to factors like 3D HetNets and TN-NTN integration, cell densification, a massive number of connections, and high data traffic. Addressing these complexities requires a combination of advanced techniques and strategies to ensure optimal network performance and seamless connectivity.

Techniques such as beamforming and mMIMO focus radio signals in specific directions to minimize interference with other devices and enhance signal quality. DSA allows devices to switch to less crowded frequency bands, reducing the likelihood of interference. Coordinated multi-point (CoMP) involves coordination among multiple base stations to manage and mitigate interference, thereby improving overall network performance. Adaptive power control adjusts the transmission power of devices based on real-time network conditions to minimize interference while maintaining communication quality. Network slicing creates isolated virtual networks tailored to specific services or user groups, reducing the risk of cross-interference. AI and machine learning algorithms predict and manage interference in real time by analyzing network conditions and user behavior, optimizing resource allocation and scheduling.

The introduction of CF architecture, combined with mMIMO and beamforming, presents both challenges and opportunities for interference management. Coordinated interference management across distributed antenna systems is important to ensure optimal performance. Additionally, ISAC and RIS can be utilized to manipulate the propagation environment, reducing interference and improving signal quality. However, their deployment also demands adaptive interference mitigation techniques.

3D HetNets and TN-NTN integration introduce additional complexity due to the diversity of propagation environments and interference sources. Coordinated interference management across different network tiers is necessary to prevent cross-tier interference. Moreover, efficient handover management and resource allocation are crucial to minimize interference during user mobility between TN and NTN.

Observing Table 8, which highlights the main techniques and spatial resources in wireless communication systems, including interference mitigation, one can note that more than 50% of the studies on RRM included in this survey considered the effects of interference on network performance and efficiency. However, half of these studies adopted the successive interference cancellation (SIC) technique as a solution to mitigate interference without providing a detailed analysis, thus addressing the problem only superficially.

Conversely, 28% of these studies developed comprehensive system models and created their own interference equations and models, such as RIS and beamforming. These models were effectively applied to optimization problems, numerical analyses, and simulations, leading to significant improvements in network performance and overall efficiency. Given this quantitative analysis and the discussion on interference mitigation, it can be concluded that interference mitigation, a critical function of RRM, is still in the early stages of research. Overcoming the numerous challenges in this domain is imperative for the successful deployment of 6G networks. Nevertheless, this analysis underscores the abundant research opportunities that lie ahead in the field of interference mitigation.

### 4.4. Spectrum Management

Spectrum management refers to the strategic allocation, utilization, and optimization of the available radio frequency spectrum to maximize the efficiency and effectiveness of wireless networks. This process involves several key activities [76,81,146,147]:

- Spectrum Allocation: Determining how the radio spectrum is divided among different wireless services, operators, and applications. Regulatory bodies often manage this aspect, ensuring that the spectrum is distributed fairly and efficiently.
- Spectrum Sharing: Allowing multiple users or services to access the same frequency band without causing harmful interference. Techniques like cognitive radio, dynamic spectrum access, and licensed shared access are employed to enable efficient spectrum sharing.
- Spectrum Reuse: Enhancing the use of the same frequency bands in different geographic areas or among different users, separated by distance, time, or frequency, to improve overall spectrum efficiency. This is often achieved through techniques like frequency reuse in cellular networks.
- Interference Management: Implementing strategies to minimize interference between users sharing the same spectrum. This involves power control, beamforming, and advanced signal processing techniques to reduce the impact of interference on network performance.
- Spectrum Efficiency Optimization: Maximizing the efficient use of the frequency spectrum while optimizing key QoS parameters within a given spectrum band. This includes refining modulation schemes, channel coding, multiple access techniques, and employing technologies like mMIMO and beamforming.

AI-enabled spectrum management emerges as a promising solution to optimize spectrum usage, mitigate interference, and enhance network performance. By leveraging AI, it is possible to develop dynamic spectrum allocation schemes, optimize resource utilization through joint resource optimization, and explore advanced technologies like RIS and terahertz communications [147].

In the context of 6G RRM, spectrum management can be significantly enhanced through data-driven approaches such as machine learning, deep learning, and reinforcement learning (RL), which offer dynamic and adaptive capabilities by learning from real-time data. These methods enable more efficient spectrum utilization by optimizing resource allocation, reducing power consumption, and maximizing throughput while addressing spectrum inefficiency through techniques like OFDMA and cognitive radio networks (CRNs). RL, in particular, is effective in managing the complexities of dynamic spectrum environments, where conflicting objectives such as power, spectrum usage, and interference must be balanced. This adaptive framework improves spectrum allocation, enhances network capacity, and ensures fairness and QoS, making it a possible approach for future 6G networks [146,148].

A comprehensive spectrum management framework for 6G networks integrates regulatory policies, advanced spectrum management systems, and cutting-edge enabling technologies. Regulatory bodies provide the foundational guidelines for spectrum allocation and usage, ensuring fair and efficient distribution. Spectrum management systems, in turn, implement dynamic and adaptive allocation strategies, including real-time RF analysis and interference management. Emerging technologies, such as massive MIMO and RIS, play a pivotal role in enhancing spectral efficiency, boosting network capacity, and supporting the complex demands of 6G networks [147].

Spectrum management has been traditionally employed to optimize radio resource utilization and ensure QoS in wireless networks. Traditionally, spectrum access was exclusively licensed, with spectrum allocated to specific users or services for defined periods. However, to enhance spectrum efficiency, dynamic spectrum access emerged, enabling unlicensed users to opportunistically access unoccupied spectrum bands. This requires spectrum awareness mechanisms to identify available spectrum opportunities through passive or active sensing techniques [149].

In the evolving landscape of 6G, dynamic spectrum management (DSM) is important to address the increasing bandwidth demands and optimize spectrum use. Traditional static spectrum management has been shifting towards a spectrum sharing model to accommodate diverse users and applications. This transition necessitates more flexible and efficient spectrum allocation strategies that can adapt to real-time conditions and user needs. The incorporation of cognitive radio technologies and AI-driven models enhances spectrum efficiency by enabling real-time spectrum access and intelligent decision-making processes [150].

The smart spectrum management (SSM) model integrates historical spectrum data with real-time observations to optimize spectrum utilization. This approach improves on conventional DSM by offering better spectrum opportunity utilization and adapting to varying spectrum environments through intelligent learning and decision-making. The SSM framework aims to bridge the gap between spectrum sensing and allocation, making use of advanced data analytics and AI to address challenges related to spectrum efficiency and resource management. As a result, the SSM model promises significant improvements in spectrum utilization, even in low signal-to-noise environments, by combining historical insights with dynamic spectrum sensing [150].

Spectrum management is increasingly challenged by the limited availability of spectrum resources due to the extensive occupation by various communication services [146,148,151]. Massive MIMO technology, which significantly increases antenna count, plays a key role in enhancing spectral efficiency by dynamically adjusting signals in both horizontal and vertical directions, reducing inter-cell interference and supporting spatial multiplexing among multiple users. Additionally, OFDM modulation is employed as the primary transmission method for high-data-rate systems due to its efficient spectrum utilization and resistance to multipath interference, further contributing to effective spectral management in 6G networks [151].

In 6G RRM, spectrum management is becoming increasingly complex with the integration of TNs-NTNs, including satellites and aerial nodes, into unified 3D networks [152,153]. The conventional “first come, first served” approach to satellite frequency and orbit allocation has led to spectrum scarcity and significant interference among large-scale satellite constellations. This inefficiency, coupled with varying satellite orbits, results in chaotic resource allocation and difficulties in providing consistent computing services. Furthermore, the dynamic nature of satellite networks, with rapidly moving satellites, exacerbates the complexity of managing spectrum resources efficiently [153].

ITU is developing recommendations to harmonize global spectrum regulations for these systems, while 3GPP is working on standards to ensure seamless connectivity across terrestrial, aerial, and satellite networks. Research is focused on enhancing data rates and capacity through techniques such as spectrum sharing and efficient duplexing, as well as managing interference between various satellite constellations and between TN-NTN systems [152]. Cognitive radio technology is being explored to address dynamic spectrum management challenges, particularly in optimizing handover procedures in congested NTNs [152,153]. To address these challenges, ref. [153] proposes collaborative deep reinforcement learning (DRL) combined with federated learning (FL) as an effective solution for spectrum management. This approach allows for dynamic and responsive spectrum allocation by utilizing AI models to adapt to interference environments and varying service demands without relying on historical data. By enabling global coordination through DRL and local learning through FL, this method enhances spectrum efficiency and reduces dependence on centralized servers, thus improving network robustness and managing interference more effectively [153].

NOMA has emerged as a promising technique to improve spectral efficiency by allowing multiple users to share the same time and frequency resources. However, NOMA’s complexity and limitations in terms of hardware and system overhead hinder its widespread adoption. To overcome these challenges, the integration of AI into multiple access schemes can improve spectrum sensing, resource allocation, interference management, and, consequently, spectrum utilization [106]. Moreover, integrating AI with emerging technologies such as ultra-massive MIMO, THz, and RIS can significantly boost spectral efficiency and support high data rates by effectively managing higher frequency bands and optimizing beamforming techniques [146].

The introduction of new frequency bands, such as mmWave and THz, offers opportunities for increased spectrum availability but also introduces new challenges related to propagation, interference, and device complexity. To address these challenges, dynamic bandwidth allocation strategies are proposed, including variable bandwidth and the use of both licensed and unlicensed spectrum. Additionally, the adoption of advanced waveforms and multiple access techniques aims to improve spectral efficiency and accommodate high traffic density. Furthermore, ISAC is explored to enhance spectrum utilization and manage the increased complexity of future networks [154].

The ISAC technology enhances spectrum efficiency by allowing the same frequency bands to be used for both radar and communication signals, which can improve energy efficiency and reduce hardware costs. This approach, especially when applied to integrated satellite–terrestrial networks, enables better spectrum utilization by integrating cognitive radio techniques that allow for the dynamic sharing of spectrum resources between satellite and TN. Such integration helps in alleviating spectrum scarcity issues and optimizing the use of available frequencies [155].

Moreover, cognitive radio technology and multi-user spectrum sharing mechanisms facilitate more efficient spectrum allocation and management by dynamically adjusting resources based on traffic demands and priority levels. ISAC systems face challenges like interference from both internal and external sources, necessitating the development of advanced anti-interference techniques and effective spectrum management strategies. The continued evolution of ISAC systems and their integration into 6G networks will require new standards and regulatory frameworks to address the complexities of spectrum sharing and interference management [155].

The topic of RF spectrum has already been briefly reviewed in Section 2.3 of this document. As AI techniques become increasingly integrated into wireless networks, recent research on spectrum management reveals a renewed focus on areas such as cognitive radio networks, cognitive radios, spectrum sensing, dynamic spectrum sharing, and similar technologies. These areas, which were intensively studied in the early 2000s, are now gaining attention again. The authors of this survey believe that the substantial research efforts invested in cognitive radios were not in vain. On the contrary, these foundational studies can be revisited and further developed to address the challenges posed by 6G systems. Spectrum efficiency and flexibility, critical in the 6G landscape, underscore the essential role of advancing cognitive radio technologies.

### 4.5. Power Control

Power and energy were the most frequently allocated and controlled resources in the RRM papers examined. These functions are often studied alongside other key RRM functions such as resource allocation, sustainability, interference management, and QoS management, highlighting their intrinsic interconnection. Power management in RRM can be broadly categorized into two distinct functions: power control and power allocation (PC&A). Power control focuses on regulating the transmission power of devices to minimize interference and optimize network performance, while power allocation involves distributing available power resources efficiently across the network to ensure optimal operation and sustainability [156,157,158].

Among the RRM studies surveyed, power allocation was employed in 42% of cases, while power control was utilized in 20%. Furthermore, PC&A in conjunction with power-domain NOMA was investigated in 27% of the examined works on RRM, which also considered any multiple access techniques in their system modeling.

PC&A is an important component of 6G RRM, intricately linked to energy efficiency. Given the substantial energy consumption of network infrastructure and the growing proliferation of battery-powered IoT devices, effective PC&A is essential for reducing operational costs, prolonging device battery life, and promoting sustainable network operations. Moreover, the potential for energy harvesting from RF signals underscores the need for intelligent PC&A mechanisms to optimize energy utilization [70].

The CF approach significantly impacts PC&A in 6G networks. By leveraging distributed antenna systems and coordinated multi-point transmission, CF networks can dynamically adjust power levels to optimize signal quality and reduce interference. This approach enhances energy efficiency and ensures consistent communication quality, even in high-density environments [34,75,76,81,113].

In 3D HetNets and TN-NTN environments, PC&A becomes more complex due to the diverse propagation conditions and interference patterns. Coordinated PC&A between terrestrial and non-terrestrial networks is important to prevent excessive interference and optimize energy consumption. Additionally, PC&A algorithms must consider the specific characteristics of different network layers and user equipment [35,40,48,81,97,109,110].

Advanced technologies such as NOMA, ISAC, mMIMO, beamforming and RIS also play relevant roles in PC&A within the scope of 6G RRM. ISAC enables more accurate and efficient power adjustments to reduce energy consumption and interference. mMIMO and beamforming technologies enhance spatial resource management by focusing power precisely where it is needed, optimizing energy use and improving communication quality. RIS technology dynamically modifies the propagation environment to enhance signal quality and reduce power requirements [33,35,40,48,75,92,105,117].

Current trends in 6G RRM research emphasize the integration of PD-NOMA with other advanced technologies, such as AI and ML, to dynamically optimize power control and allocation. These trends aim to enhance the adaptability and efficiency of PD-NOMA under diverse network conditions and user scenarios, improving overall network performance and user experience. Despite its advantages, PD-NOMA faces challenges such as managing computational complexity, addressing security concerns, and ensuring fairness among users with diverse channel conditions. The use of SIC, while advantageous for decoding overlapping signals, adds to the complexity of RRM. However, the opportunities for innovation in PD-NOMA, including improved connectivity and energy efficiency, position it as a pivotal technology in achieving the ambitious goals of 6G networks.

### 4.6. Mobility Management

Mobility management involves handling user movement within the network to ensure seamless connectivity and service continuity. This function is important for maintaining QoE as devices move across different network areas [51,56]. CF and CL approaches have the potential to provide ubiquitous coverage and consistent service quality, regardless of user location. This results in smoother handovers, reduced latency, and improved network reliability by dynamically reallocating resources to follow the user’s movement. Consequently, users experience uninterrupted service and consistent performance, even in highly mobile scenarios [51,112].

Additionally, the ISAC techniques enable the network to accurately determine the location of users. This precise location awareness significantly enhances mobility management by allowing the network to proactively adjust resources and optimize connectivity based on real-time user positions. ISAC’s ability to provide detailed location data supports more efficient handover processes, minimizes service disruptions, and enhances overall network performance [92,118,119].

The integration of TN and NTN reshapes mobility management within 6G RRM. This integration extends network coverage to remote and underserved regions, posing significant challenges in handover processes as users transition between TN and NTN. Efficient mobility management must address these transitions to minimize service disruption and maintain consistent QoS despite the varying performance characteristics of TN and NTN segments. Key issues include optimizing handover algorithms, managing latency and resource allocation, and ensuring seamless connectivity as users move between different network domains [56,115,137,138,139].

Future advancements in mobility management for TN-NTN integration will benefit from the development of sophisticated algorithms and the integration of ML and AI. These technologies can enhance predictive capabilities for user movement, optimize dynamic handover processes, and adjust network parameters in real time. Collaborative strategies between TN and NTN operators and standardized protocols will be relevant for achieving seamless integration and efficient resource utilization, thereby improving overall network performance and user experience in the 6G era [56,115,137,138,139].

When analyzing the articles on RRM investigated for this research, it is notable that only 8% of the works addressed the RRM function of mobility management (see Table 8). In the context of the 6G ecosystem, it is notable that significant research is still needed in this area. The limited focus on mobility management highlights a gap in the current literature and suggests ample opportunities for further exploration.

### 4.7. Communication Mode Selection

In 6G networks, the selection of the optimal communication mode is a critical RRM function, especially given the advancements introduced by CF networks. These innovative approaches fundamentally alter the traditional communication framework by shifting from fixed base stations to a network of distributed access points. Consequently, selecting the appropriate mode of communication—whether it be direct base station communication, device-to-device (D2D) interactions, or connections through TN and NTN—becomes pivotal for ensuring efficient and reliable connectivity. This choice must account for factors such as network density, mobility, and the specific requirements of user equipment [159].

The introduction of 3D HetNets further complicates communication mode selection by incorporating a multi-dimensional network structure that includes various tiers of connectivity. This complexity necessitates dynamic and context-aware mode selection strategies to optimize performance across different layers of the network. For instance, mMIMO and beamforming technologies can enhance the effectiveness of selected communication modes by improving coverage and capacity. Additionally, RIS can further refine communication by enabling adaptive signal processing and improving link reliability. The integration of these technologies must be considered when determining the most suitable communication mode for different scenarios within a 3D HetNet [159].

In the context of TN-NTN integration, communication mode selection involves choosing modes that facilitate efficient handovers, minimize latency, and optimize resource usage across both terrestrial and satellite networks. The ability to dynamically select and switch between different communication modes based on real-time network conditions and user needs is essential for maintaining high-quality service and network efficiency. ISAC plays a significant role here by providing real-time environmental and network data that informs the optimal communication mode selection [159].

The dynamic formation of clusters is another important aspect of communication mode selection in 6G networks. Effective cluster formation helps to balance network load, enhance resource allocation, and improve overall network performance. Therefore, communication mode selection should be integrated into the broader clustering strategy to ensure that it supports the formation of efficient and functional network clusters [159].

Table 9 provides an overview of studies on 6G RRM, emphasizing critical functions such as communication mode selection, admission control, and user association. Notably, ≈50% of the analyzed works specifically address the communication mode selection function. In the reviewed articles, communication mode selection was always used in conjunction with other RRM functions, such as resource allocation. This combination was generally applied within two primary system models: D2D communication, which accounted for ≈4% of the studies, and integrated communication between TN and NTN. This integration highlights the evolving complexity and interdependency of RRM functions in 6G networks, necessitating advanced coordination strategies to optimize overall network performance. Moreover, it highlights the significant challenges involved in managing radio resources in future wireless communication networks.

### 4.8. Admission Control

Admission control in 6G goes beyond simple acceptance or rejection of connection requests. It involves a comprehensive evaluation of network conditions, QoS requirements, and the selection of the most suitable communication mode. This function is necessary to maintain network stability and ensure that existing connections meet their QoS requirements. Given the limited and costly nature of network resources, admission control must operate efficiently to balance resource allocation and service quality [159,160,161,162].

In the context of RRM, CF, TN-NTN integration and 3D HetNet, admission control is integral to the dynamic formation of clusters. Clusters form based on user density, mobility patterns, and service demands. Admission control determines which users can join a cluster and the communication mode within the cluster. This decision influences load balancing, interference minimization, and resource optimization, thereby enhancing overall network performance [159].

To harness the strengths of both centralized and distributed control, a hybrid communication mode is likely to be the most promising approach. This involves combining centralized decision-making for critical functions like initial cluster formation and user admission policies with distributed control for real-time adjustments and local optimization. By carefully defining the roles and interactions between centralized and distributed entities, it is possible to achieve an optimal balance between performance, scalability, and reliability [159,160,161,162]. Admission control in 6G is also expected to leverage technologies like mMIMO, beamforming, RIS, and ISAC to ensure that resources are allocated effectively [45,159].

Based on Table 9, the admission control function was addressed in less than 20% of the articles on RRM. This indicates that most research assumes the network can support all incoming connections, implying an infinite load capacity. Consequently, the QoS indicators in these studies are considered to depend solely on channel conditions and interference, primarily from the signal-to-interference-plus-noise ratio (SINR). However, in reality, resources such as transmission power, frequency spectrum, processing and storage capacity, antennas, and time slots are limited and costly. The lack of consideration for these constraints in admission control can lead to an unrealistic evaluation of network performance and QoS in practical scenarios.

The authors of this work suggest significant research opportunities for admission control in 6G networks within the context of 6G RRM. They emphasize the importance of considering resource limitations and costs, such as power, frequency, and processing capabilities, to provide a realistic assessment of network performance and QoS. Future research should focus on developing dynamic admission control algorithms that adapt to varying network conditions and prioritize connections based on QoS requirements and current network load.

Additionally, the authors propose multi-dimensional resource management frameworks that account for various resource types simultaneously to optimize network performance. They advocate for the use of realistic simulation environments and real-world testing to validate proposed admission control mechanisms, bridging the gap between theory and practice. Furthermore, integrating advanced technologies like mMIMO, beamforming, RIS, and ISAC with admission control can enhance the adaptability and efficiency of these mechanisms in 6G networks.

### 4.9. User Association

User association in 6G networks involves the process of linking user equipment with a suitable base station or AP to optimize network performance and resource allocation. Traditionally, this function has relied on factors such as signal strength, but the evolving complexity of 6G networks necessitates more advanced criteria. SINR, load balancing, and energy efficiency are increasingly important considerations. Effective user association plays a significant role in managing network interference and maintaining seamless communication, which are important for meeting the performance and QoS requirements of 6G [159,163,164].

In 6G networks, the role of user association becomes more prominent due to the demand for high data rates, low latency, and extensive connectivity. The dynamic nature of 6G, marked by frequent changes in network topology, user mobility, and traffic patterns, calls for adaptive user association algorithms. Emerging technologies like CF networks, mMIMO, and RIS introduce new challenges that require user association mechanisms to be flexible and responsive. Proper user association in 6G networks will be central to unlocking the full capabilities of these technologies [35,112,159].

The effectiveness of user association algorithms can be assessed using KPIs such as system throughput, energy efficiency, user fairness, and load balancing. To address the complexities of 6G, these algorithms need to account for user mobility, channel dynamics, and varied QoS requirements. The incorporation of ML techniques can enhance user association by enabling real-time adaptations to fluctuating network conditions, which is important for sustaining optimal performance in a 6G environment [34,112,159].

User association is also integral to cluster formation in 6G networks. Clustering UEs based on factors like proximity, channel conditions, and traffic patterns allows for more efficient resource allocation and interference management. This approach, combined with advanced RRM functions such as power control, beamforming, and interference coordination, can significantly enhance network performance. By tailoring RRM strategies to specific clusters, networks can achieve more efficient resource utilization, ultimately leading to a better user experience in 6G [34,80,112,159].

A comprehensive review of the RRM-related literature reveals that user association is incorporated into resource management optimization in approximately 24% of studies (see Table 9). While user association is consistently explored in conjunction with other RRM functions, it is often treated as a secondary component. Notably, no research was identified that simultaneously addresses user association, communication mode selection, and admission control as core elements of RAN management within the RRM framework. Moreover, the literature lacks a unified approach to RRM that integrates key 6G enablers, such as heterogeneous networks, CF architecture, TN-NTN integration, dynamic clustering, mMIMO, beamforming, and intelligent surface-assisted communications. This gap highlights the need for a more integrated RRM paradigm to fully exploit the potential of 6G networks.

Ultimately, the effective implementation of communication mode selection, admission control, and user association within 6G networks will be instrumental in realizing the full potential of 6G technologies. This will not only enhance network performance and user experience but also pave the way for innovative applications and services that leverage the advanced capabilities of 6G systems [165,166].

### 4.10. Congestion Control

Congestion control, load balancing, and QoS management within RRM are intrinsically tied to the admission control function. New traffic can only be admitted to a network with constrained resources if it can be accommodated without compromising the QoS of existing traffic or violating any service level agreement conditions. Effective admission control algorithms are valuable for preventing network congestion, minimizing the probability of dropped connections, and optimizing network resource utilization [159,167].

Congestion control in 6G networks is increasingly important given the anticipated exponential growth in data traffic driven by advanced applications, including IoT, vehicular communication, and immersive media. The management of this congestion is important for maintaining QoS across diverse and resource-intensive services [168,169]. Congestion, which occurs when demand for network resources surpasses supply, can lead to significant performance degradation, including increased latency, packet loss, and reduced throughput. This makes effective congestion control a relevant aspect of 6G RRM, as it ensures the efficient use of network resources while maintaining service quality for end users [170,171].

Traditional congestion control mechanisms, such as those explored in earlier generations of wireless networks, relied heavily on static approaches like congestion pricing, which adjusts service costs based on network load. Although these methods provided a foundational approach to managing congestion, they often failed to account for the dynamic and heterogeneous nature of modern wireless networks, where varying user demands and network conditions require more adaptive solutions. For instance, congestion pricing, while effective in some contexts, does not adequately address the needs of users who require consistent, low-latency communication, such as those in vehicular networks or smart factories where even minor delays can have significant consequences [168,170,171].

Emerging techniques in congestion control are shifting towards more intelligent and adaptive methods. The use of DRL, for example, represents a significant advancement in the ability to manage congestion dynamically. DRL-based congestion control mechanisms can learn from the network environment, adapting to changes in real time and making decisions that optimize resource allocation while minimizing congestion. This is particularly relevant in 6G environments characterized by high mobility, such as vehicular networks or unmanned aerial vehicles, where network conditions can change rapidly and unpredictably [168,170,171].

In the context of CF mMIMO and RIS, congestion control must also evolve to manage the complexity of these systems. CF networks pose unique challenges for congestion control as the distributed nature of these networks can lead to increased coordination overhead and potential congestion in certain network segments. RIS, on the other hand, can be used to dynamically reconfigure the radio environment, potentially mitigating congestion by optimizing the propagation conditions and steering traffic away from congested areas [171].

Another critical aspect of congestion control in 6G is its integration with other RRM functions, particularly in the management of multi-connectivity and NTN. As 6G networks are expected to leverage a combination of terrestrial and satellite links, managing congestion across these diverse paths will be essential. The ability to dynamically switch between different communication modes or adjust the use of NTN links based on congestion levels will be important for maintaining service quality in scenarios where traditional congestion control mechanisms might fail due to the highly variable nature of satellite communication links [168,170,171].

Finally, as 6G networks move towards fully autonomous and self-optimizing systems, the role of congestion control will likely extend beyond traditional traffic management to include predictive and proactive measures. This might involve the use of AI to anticipate congestion based on historical data and current network trends, enabling preemptive actions such as traffic rerouting or resource reallocation before congestion even occurs. Such an approach would represent a significant departure from the reactive methods of the past, offering a more resilient and efficient way to manage the increasingly complex and dynamic traffic patterns expected in 6G networks [168,170,171].

A comprehensive review of the RRM literature reveals a significant research gap in the explicit consideration of congestion control. Despite the anticipated exponential growth in traffic, proliferation of devices, and complex service requirements of 6G networks, only 4% of examined studies prioritized congestion control as a secondary RRM function. The intricate interplay of these factors, coupled with the introduction of new technologies like mMIMO and RIS, will exacerbate congestion risks. To address this, the development of dynamic, intelligent, and adaptable congestion control algorithms is imperative. This presents a substantial research opportunity to enhance 6G network performance and user experience.

### 4.11. Load Balancing

Load balancing is responsible for distributing network traffic evenly across available resources to prevent congestion and optimize performance. By evenly distributing workloads, load balancing helps to enhance resource utilization, minimize delays, and maintain consistent QoS for users across various network segments. This function is particularly significant in HetNets, where the integration of multiple radio access technologies and the dynamic nature of traffic require sophisticated mechanisms to ensure that no single node is overwhelmed while others remain underutilized. The importance of load balancing becomes even more pronounced with the increasing complexity of 6G networks [73,97,112,172].

In indoor environments, particularly in heterogeneous femtocell networks, load-balancing mechanisms are important for mitigating the effects of uneven user densities and temporal or spatial traffic fluctuations. Self-organizing networks (SON) introduce intelligent mechanisms to automatically tune network parameters, redistributing users from overloaded cells to less congested neighboring cells. This approach enhances network capacity and ensures consistent QoS for users, as outlined in the study of load-balancing mechanisms for indoor heterogeneous femtocell networks [173].

In CF and CL network architectures, where users are served by a distributed set of APs rather than a single base station, load balancing plays a pivotal role in optimizing resource distribution. By dynamically associating users with the optimal set of APs based on real-time conditions, load balancing helps to prevent bottlenecks and ensures seamless connectivity even in densely populated areas. Techniques such as user association and dynamic resource allocation are employed to adapt to fluctuating network demands, thereby enhancing the overall performance of the network. The integration of these approaches within cell-free networks facilitates efficient load distribution, enabling the network to scale effectively while maintaining high levels of user satisfaction [112,172,173,174].

In the context of TN-NTN integration, load balancing becomes a complex but essential task. The variability in performance between terrestrial, aerial, and satellite segments demands adaptive load-balancing strategies that can dynamically route traffic based on real-time conditions. For instance, in integrated satellite–aerial–terrestrial networks, load balancing must account for factors such as atmospheric conditions, mobility, and the varying capabilities of different network segments. This is where advanced technologies like network slicing, ML and AI come into play, offering the ability to predict and adjust to changing network conditions, thus ensuring optimal traffic distribution and maintaining service continuity across all segments [47,97,175,176].

Load balancing is also intertwined with the deployment of mMIMO and beamforming. By dynamically adjusting beam directions and power levels, mMIMO and beamforming can facilitate more effective load distribution across the network. Additionally, the use of RIS enhances load balancing by manipulating the propagation environment to favor balanced traffic distribution, reducing the likelihood of bottlenecks and improving overall network efficiency [105,110,112,176].

In outdoor and indoor scenarios, load balancing is often integrated with admission control mechanisms to manage the traffic load across base stations. This process typically involves the handover of user terminals between adjacent cells, ensuring an even distribution of traffic while maintaining user QoS. In OFDMA-based relay networks, load balancing is also linked to the distribution of OFDM subcarriers among cell nodes, which is a common estimate of traffic load. A balanced distribution of subcarriers reduces packet processing delays at regenerative relays and ensures fair utilization of energy resources, particularly in networks employing battery or solar-powered relay stations [172,177].

Quantitative analysis of the surveyed works on RRM reveals that only 10% of the studies marginally consider load balancing. This finding underscores the limited focus on this critical function, despite its importance in managing network resources efficiently. Moreover, only 4% of the studies investigated the combined analysis of load balancing and admission control, even though these functions are closely interconnected. This gap highlights the tendency in current research to address RRM functions in isolation, neglecting the synergies between them.

The isolated approach prevalent in these studies not only limits the understanding of the real impact of RRM functions on the performance and efficiency of 6G systems but also overlooks the broader implications of integrating these functions with enabling technologies for future 6G networks. By focusing on narrow and specific system models, researchers miss the opportunity to explore how these RRM functions can be optimized in conjunction with key 6G technologies. This fragmented perspective hinders the development of comprehensive RRM strategies that are important for the efficient operation of 6G networks.

In conclusion, there is a significant need for more integrated research on RRM functions to support the design, standardization, and specification of 6G. The current research landscape presents both challenges and opportunities for advancing the understanding of how RRM functions can be effectively integrated and optimized within the broader context of 6G technologies. Addressing these gaps will be essential for overcoming the challenges and unlocking the potential of 6G systems.

### 4.12. QoS Management

QoS management focuses on delivering consistent and reliable service quality, which is assessed through specific KPIs. These KPIs, derived from QoS parameters, provide quantifiable metrics to evaluate and optimize the network’s performance. By continuously monitoring and adjusting resources based on these KPIs, QoS management ensures that 6G networks can meet diverse and demanding service requirements across various applications [144,178,179].

QoS management involves ensuring that different types of services receive appropriate levels of performance based on their specific requirements. While traditional QoS metrics, such as throughput, latency, and packet loss, remain essential, the relative importance of these parameters may shift. Additionally, 6G introduces new QoS dimensions, including reliability, security, mobility, positioning, beamforming, sensing, and sustainability, to ensure the delivery of diverse services with varying performance requirements [144,180,181].

In the quantitative analysis of the QoS management function, every study on RRM reviewed in this survey considered at least one QoS metric for optimization within a specific system model. Data rate maximization emerged as the most frequently investigated QoS metric, appearing in 30% of the RRM studies. For the anticipated new QoS metrics in 6G systems, sustainability—linked to minimizing energy consumption or maximizing energy efficiency—was the most explored, representing 18% of the RRM research covered in this survey.

While the precise service categories for 6G networks remain under development, as detailed in Section 2, the RRM function of QoS management has begun to be explored based on anticipated 6G use cases and extrapolated from 5G service requirements. Consequently, researchers have made informed inferences about potential QoS metrics necessary to support emerging 6G applications. This approach, though preliminary, provides a foundational understanding of QoS management challenges and opportunities in the context of 6G network design. Future research should expand the scope of QoS metrics to encompass a wider range of performance indicators and explore innovative strategies to optimize network performance while minimizing energy consumption.

### 4.13. Beamforming and Beam Management

Beam and beamforming management (BBM) are pivotal components of 6G RRM. Beam management involves coordinating and directing energy patterns, or beams, to target specific users or areas. In the dense device environments of 6G networks, this task becomes increasingly complex. Advanced algorithms are important for efficiently allocating beams and minimizing interference. Beamforming management, which involves adjusting the phase and amplitude of signals to shape these beams, complements beam management by enhancing signal quality, coverage, and reducing interference. Together, these functions are essential for meeting the demands of 6G networks, particularly when supporting mMIMO and RIS that heavily rely on precise beam control.

In the evolving landscape of 5G and 6G wireless communication systems, BBM is becoming increasingly intricate due to the use of MIMO antenna arrays and the adoption of mmWaves and THz. The complexity introduced by user mobility and the sheer number of antennas necessitates advanced approaches to ensure optimal system performance. AI, particularly ML, offers promising solutions to streamline these processes, reducing the overhead typically associated with BBM tasks such as beam tracking and beam selection. By leveraging context information, AI-guided BBM can enhance the efficiency of beam selection and optimize beamforming mechanisms, potentially achieving significant time reductions in these operations. The integration of AI into BBM is especially relevant for 6G networks, which demand greater agility, adaptability, and environmental modeling capabilities. Despite the potential benefits, the literature highlights the need for further research to refine AI techniques for more dynamic and effective beamforming management in these advanced networks [182].

Deep learning offers significant advantages in BBM by modeling complex nonlinear factors, such as user mobility and channel variations, and effectively handling the high-dimensional features of propagation scenarios. For instance, deep learning techniques are employed for high-resolution beam prediction, which facilitates accurate angle of arrival (AoA) and angle of departure (AoD) estimation, important for minimizing beam-training overhead. Additionally, side-information-aided beam selection utilizes deep learning to assimilate environmental features, enabling more efficient beam alignment by leveraging the shared characteristics of low-frequency and mmWave channels. Furthermore, deep learning models such as Long Short-Term Memory networks and transformers are applied in beam tracking to handle sequential data and capture both short-term and long-term dependencies, which are critical for adapting to time-varying channels and maintaining optimal beamforming direction in real time [148].

RL and DRL further enhance BBM by allowing communication systems to learn and adapt dynamically to the changing wireless environment. RL models continuously interact with the environment, refining beamforming strategies to maintain optimal communication links despite variations in user positions, channel conditions, and network loads. DRL, which combines RL with deep learning techniques, uses deep neural networks to approximate value functions or policies, offering a scalable solution to the challenges of BBM in complex and dynamic 6G networks. However, these data-driven approaches also introduce challenges, such as the need for high-quality datasets, energy efficiency concerns, and the complexity of managing beams for multiple high-mobility users. Addressing these challenges will be important for the successful implementation of BBM strategies in 6G networks [148].

Effective BBM is relevant in 6G systems, particularly with the integration of mMIMO and ultra-mMIMO technologies, as well as the utilization of THz frequencies. These technologies enable advanced beam steering and beamforming capabilities, essential for meeting the demands of AI-driven applications requiring high reliability, low latency, and high data rates. The precise alignment of beams between the access point and user equipment is necessary for achieving maximum antenna array gains and overcoming challenges like high path loss and rapid channel variations. However, this precision also introduces latency in link establishment, necessitating optimal beam sweeping and alignment strategies to maintain performance. Additionally, in multi-user (MU) mMIMO systems, low-complexity solutions are required to reduce transmission power while minimizing inter-user interference, ensuring that beamforming remains efficient even in high-mobility environments where smooth handover is critical. This requires robust beam tracking and selection techniques to maintain QoS in dynamic scenarios [148,183,184].

In the 6G networks, RIS emerges as a transformative technology for enhancing beamforming management, providing efficient and reliable wireless signal transmission. RIS utilizes smart beamforming methods, controlled by an electronic circuit, to adjust the reflecting angle of its elements, directing beams towards multiple users with improved signal intensity proportional to the number of elements involved. This approach allows RIS to achieve similar beamforming gains as mMIMO systems but with lower power consumption. By targeting specific directions or users, RIS can significantly increase wireless channel capacity and enhance overall system performance, particularly for devices that need to offload computational tasks to edge nodes with minimal energy use [185,186].

The integration of RIS with mMIMO technology in 6G networks further optimizes beamforming management by expanding spatial degrees of freedom and increasing spectrum efficiency. Although mMIMO involves high energy costs and hardware expenses, combining it with RIS reduces these challenges, enabling more efficient resource allocation and beamforming designs. Additionally, RIS-aided beam tracking is critical for handling mobile user equipment in dynamic environments. Through accurate sensing capabilities, RIS can gather data on UE position, environmental layout, and mobility patterns, facilitating precise beam tracking and ensuring optimal performance in 6G networks [185,186].

The integration of sensing into BBM, particularly through ISAC, allows for a more environmentally aware communication system. Sensing technologies provide critical information about the surroundings, such as the geometry of scatterers and the location and movement of users, which can be used to predict and track beams, avoiding the need for extensive beam training. This approach is especially beneficial in highly dynamic scenarios, such as vehicular communication, unmanned aerial vehicles, and high-speed trains, where channels are constantly changing, and rapid reconfiguration is required. Sensing-assisted BBM thus reduces overhead and enhances the adaptability of mmWave and THz communication systems in 6G [187].

Finally, the management of THz beams in 6G wireless networks presents unique challenges, particularly due to the need for much narrower beams to compensate for higher propagation losses compared to mmWave. This requires precise beam training that accounts for both the direction and distance between the transmitter and receiver, particularly in near-field scenarios where traditional far-field approaches fail. The current state of research on THz BBM is still nascent, with most studies focusing on far-field scenarios. There is a pressing need for more in-depth exploration of near-field effects and the development of corresponding BBM strategies to ensure the effectiveness of THz communication systems in 6G networks [187].

A quantitative analysis of the surveyed RRM research reveals a significant gap in the investigation of BBM functions. While 16% of studies addressed BBM, none specifically focused on CF networks. Only 6% of BBM studies explored the integration of TN-NTN or 3D HetNet architecture. Additionally, the combination of RIS and BBM was investigated in just 4% of RRM studies, and ISAC was considered in only 4% of BBM-related research. Given the critical role of BBM in 6G RRM, further research is needed to address these knowledge gaps.

Table 10 highlights the primary techniques and technologies considered in the RRM studies analyzed for this survey. A quantitative analysis of the surveyed RRM research reveals a strong emphasis on diversity techniques. These techniques, which include techniques like OFDM, spatial multiplexing, and time division multiplexing, were employed in 30% of the studies to enhance network performance and efficiency. Following this, the reviewed studies utilized AI techniques or technologies (38%), NOMA (23%), SIC and beamforming (40%), and MIMO/mMIMO (23%) as significant methods to achieve these goals. Conversely, crucial technologies such as ISAC were considered in only 10% of the studies, network slicing in 12%, RIS in 6%, and THz technologies in a mere 3% of the works investigated on RRM for this survey. This distribution suggests that while some advanced techniques are gaining traction, others, critical to the envisioned capabilities of 6G networks, remain underexplored.

In the context of 6G RRM, system modeling has primarily focused on cluster formation (28%), adaptations and modifications within RANs (24%), heterogeneous networks in three dimensions, and TN-NTN integration, as well as CF or CL architectures (32%). However, none of the studies examined a comprehensive system model anticipated for 6G, which should incorporate a wide frequency spectrum, CF or CL architecture, TN-NTN integration, and 3D heterogeneous networks. Although the majority of the selected RRM works address 6G networks, they often overlook crucial techniques and technologies envisioned for future systems. Furthermore, while these studies frequently incorporate individual important 6G techniques, they do not explore their combined impact on network performance and efficiency within a complex 6G system model. This gap highlights the need for further research to understand how these techniques will interact and affect 6G network performance when integrated into comprehensive system models.

### 4.14. Security

Security refers to protecting the network and its data from unauthorized access, theft, damage, or disruption. It involves measures to prevent malicious activities like hacking, eavesdropping, and data breaches. Security is about ensuring the confidentiality, integrity, and availability of information and network resources [5]. The main focus areas are:

- Confidentiality: Ensuring that sensitive data remains private and inaccessible to unauthorized parties.
- Integrity: Protecting data from unauthorized modification or corruption.
- Availability: Ensuring that the network and its resources are accessible to authorized users when needed.
- Authentication and Authorization: Verifying the identity of users and controlling their access to network resources.
- Non-repudiation: Preventing users from denying their actions or transactions.
- Access control: Restricting access to network resources based on user roles and privileges.
- Encryption: Using cryptographic algorithms to scramble data, making it unreadable to unauthorized parties.
- Intrusion detection and prevention: Identifying and blocking malicious activities that attempt to compromise the network’s security.
- Risk management: Assessing and mitigating security risks to the network.
- Compliance: Adhering to relevant security regulations and standards.

The last two RRM functions to be introduced—security and sustainability—are relatively new, set to be fully integrated into future 6G networks, despite having been widely discussed since the early studies of 5G networks, which are now being implemented. Security, as an RRM function, is particularly critical, complex, and often controversial. For instance, 6G networks aim to achieve a reliability KPI of 99.999999999%, a target that falls under the security function within RRM. However, such a global indicator may not fully address the diverse security needs and expectations of network users. Indeed, to the best of our knowledge, no existing metric comprehensively captures all aspects of security required for this function. Developing comprehensive and effective security indicators is an important area of research for ensuring the robustness and resilience of 6G systems.

In typical 6G network system models, the security function is not merely an isolated component; it underpins the entire implementation and risk mitigation strategy across all other RRM functions and enabling technologies [68]. The IMT-2030 framework emphasizes the integration of security and resilience as fundamental principles in the design, deployment, and operation of next-generation networks. The system’s ability to maintain operations and quickly recover from disruptive events is critical for achieving broader societal and economic objectives.

The shift towards more centralized yet increasingly fragmented technology ecosystems poses additional challenges for 6G security, as with the centralization of data and services, the risk of concentrated attacks increases. The deployment of AI in network automation and management introduces both opportunities and risks, necessitating stringent security protocols to manage AI-related vulnerabilities. This situation highlights the critical need for adaptable security frameworks that can respond to the dynamic nature of 6G networks. The anticipated deployment of 6G in the 2030s requires that foundational security principles be established now, particularly as new technologies such as ISAC and virtualization reshape the network landscape. The work of standardization bodies like ETSI is instrumental in defining these security standards, ensuring they are comprehensive and globally interoperable [68].

The ETSI Technology Radar highlights the increasing importance of integrating security into AI-driven ICT systems, particularly within the context of 6G RRM. Security-by-design is a key focus, ensuring that AI systems are not only efficient but also secure, protecting fundamental human rights such as privacy and self-determination. This approach is important for maintaining security across various layers of the network, from RAN to core network capabilities, especially as AI becomes integral to network automation and management. The standardization process emphasizes the development of interoperable security frameworks to safeguard privacy and cybersecurity. Additionally, the role of AI in autonomic networks and SONs necessitates robust security measures, given the potential vulnerabilities associated with AI-driven processes. The continued evolution of AI within ICT standards and its deep impact on security underscores the need for ongoing consideration and adaptation of security practices over time [68].

Distributed ledger technologies (DLTs), often referred to as blockchains, offer promising applications in enhancing the security of RRM within complex network environments like CF networks, TN-NTN integration, and 3D HetNets. These technologies provide robust security features by ensuring tamper resistance and enabling trust without reliance on central authorities. DLTs, particularly in permissioned models, allow for controlled participation, improving security in mission-critical scenarios. The cryptographic and distributed nature of DLTs makes it difficult to alter data once stored, providing a secure foundation for managing transactions and interactions across various 6G network components, including mMIMO, beamforming, and RIS. Additionally, DLTs can help secure ISAC systems by ensuring that data exchanged within these networks remains trustworthy and tamper-proof. However, challenges such as interoperability, scalability, and energy consumption need to be addressed to fully leverage DLTs for 6G security, particularly in IoT-intensive applications where decentralized models are crucial. These challenges, along with potential regulatory compliance issues, highlight the need for further research and standardization to integrate DLTs effectively into 6G RRM frameworks [68].

The integration of dynamic data into wide-area control loops for optimizing various processes—ranging from production to logistics and public health—introduces new security challenges. Inaccurate or compromised data can severely disrupt these control loops, leading to vulnerabilities in critical network operations. This concern extends to AI-driven systems and robotics, where data poisoning or inaccuracies can result in significant security breaches, especially in ISAC systems. The reliance on real-time data from diverse sources, including IoT devices and satellite imagery, heightens the need for robust security measures to protect the integrity and reliability of data across the entire 6G ecosystem. As dynamic data becomes increasingly embedded in 6G RRM functions, ensuring its accuracy and protecting it from tampering are essential for maintaining secure and resilient networks [68].

Quantum computing, with its ability to process information in fundamentally new ways, poses a significant threat to existing public-key cryptography, which underpins the security of current communication networks. The computational power of quantum computers could easily break widely used cryptographic schemes like Rivest-Shamir-Adleman and Elliptic Curve Cryptography, thereby compromising the confidentiality of both real-time and stored information. This vulnerability necessitates the development of quantum-safe cryptography, which aims to create algorithms resistant to attacks from both classical and quantum computers. Additionally, quantum key distribution offers a promising alternative for secure communications, as its security is based on the principles of quantum mechanics rather than computational complexity. As 6G networks evolve, incorporating quantum-safe technologies will be important to maintaining the integrity and security of information across advanced infrastructures envisioned [68].

The integration of robotics and autonomous systems (RAS) raises significant security concerns, particularly as these systems increasingly rely on advanced wireless communication technologies. The growing deployment of RAS across various sectors, such as precision farming, autonomous driving, and healthcare, introduces new vulnerabilities that must be addressed to ensure the secure operation of these systems. As RAS becomes more autonomous, the potential for cyberattacks targeting its communication systems, embedded intelligence, and machine learning capabilities increases. Ensuring the security of RAS in 6G networks involves safeguarding the integrity of data communication, protecting against unauthorized access, and mitigating risks associated with AI-driven decision-making. The emphasis on wireless communication security, safety, and collaboration with other standards organizations is essential to maintaining robust security frameworks as RAS becomes more pervasive in 6G-enabled applications [68].

Through quantitative analysis of the researched RRM studies, it was found that only 6% of the selected works addressed the security function within the context of 6G networks. Of these, a mere 4% integrated the security function with the ISAC technique. This underscores a significant research gap in the area of security for 6G networks. To address this deficiency, the development of robust security specifications, indicators, standards, and policies is imperative. These guidelines will facilitate the creation of innovative algorithms, functions, measures, techniques, technologies, and security devices tailored to the unique challenges of 6G networks. Currently, there is a reliance on competent bodies to establish the necessary frameworks for 6G security. Researchers are anticipating the availability of standardized security tools, including algorithms, functions, APIs, libraries, and ready-to-use hardware, in line with the service-based network architecture paradigm.

### 4.15. Sustainability

Sustainability is framed as a holistic concept encompassing environmental, social, and economic dimensions. The 6G architecture is designed with sustainability at its core, aiming to meet current needs without compromising future generations’ ability to do the same. This involves both the sustainability of 6G technologies themselves and the role of 6G in supporting broader societal sustainability goals. From an environmental perspective, 6G networks must address energy use, material consumption, and the associated carbon footprint. The importance of reducing energy consumption and transitioning to renewable energy sources is underscored, particularly as the ICT sector currently contributes approximately 1.4% of global carbon emissions, with a significant portion stemming from user devices. The integration of life cycle assessment methodologies is important for evaluating the environmental impact of 6G networks across their entire lifecycle, from material extraction to end-of-life disposal, ensuring a comprehensive approach to sustainability [3].

To achieve sustainable 6G networks, key technological enablers such as disaggregated and virtualized RAN and energy-efficient resource allocation strategies are essential. These technologies aim to optimize the use of communication, computation, and storage resources, thereby enhancing energy efficiency across heterogeneous network environments. The development of energy-neutral devices, supported by wireless power transfer and energy harvesting, further contributes to sustainability by reducing the reliance on batteries and minimizing environmental impact. Moreover, cloudification and the reuse of common cloud components are anticipated to play a significant role in the 6G architecture, enabling more efficient and sustainable network operations [3].

Sustainability in the environmental domain involves minimizing greenhouse gas emissions and other environmental impacts throughout the lifecycle of both networks and devices. The focus is on improving energy efficiency, reducing energy consumption, and promoting resource optimization. This includes efforts to extend the longevity of equipment through repair, reuse, and recycling, aligning with the principles of a circular economy. Energy efficiency, quantified as the amount of information transmitted per unit of energy consumption, is highlighted as a crucial metric, with improvements expected to accompany capacity increases to ensure minimal overall power consumption [5,68]. It is worth mentioning that sustainability in 6G encompasses not only power efficiency and low carbon footprints, but also resource consumption reduction, recycling practices and sustainable materials development. As an RRM function, sustainability presents a complex and sensitive challenge within the ecosystems of future 6G networks due to the broad scope. However, energy efficiency discussed in Section 2 remains the sole indicator currently associated with this function.

While energy efficiency is undeniably crucial, sustainability extends far beyond this single metric. As with the RRM security function, there is a pressing need for relevant authorities to establish comprehensive specifications, standards, and policies for the RRM sustainability function. These efforts will be important for the successful deployment of 6G networks.

The authors of this work believe that additional resources and indicators will be developed in the interim, allowing sustainability to evolve into a robust and quantifiable RRM function. This evolution will align with the broader scope of sustainability, supporting the achievement of the United Nations’ SDGs.

From the quantitative analysis of Table 10, one can note that 10% of the RRM studies reviewed in this survey focused on wireless powered communication networks (WPCN), specifically from the perspective of the RRM sustainability function. The primary techniques explored in these studies include energy harvesting, wireless power transfer (WPT), simultaneous wireless information and power transfer (SWIPT), and backscatter communication. These technologies are integral to enhancing the energy efficiency and sustainability of 6G networks, aligning with the broader goals of minimizing energy consumption while maintaining robust communication capabilities.

## 5. Optimization Strategies

Considering the selected articles on 6G RRM, 94% formulated optimization problems for radio resource allocation. The remaining studies developed customized algorithms designed to optimize specific objectives, such as enhancing network performance or minimizing resource usage. These constraints often include factors such as bandwidth and power limitations, interference thresholds, and QoS requirements, reflecting the complex trade-offs inherent in resource allocation for 6G networks [189].

Optimization problems can be broadly classified based on several criteria, each influencing the choice of appropriate solution methodologies due to their distinct characteristics and challenges. Classifications consider: the nature of variables (continuous, discrete, integer, binary); the number of objectives (single-objective or multi-objective); the problem structure (linear, quadratic, non-linear, combinatorial); the function behavior (convex or non-convex); the type of constraints (equalities; inequalities, integrity, non-linearities); the computational complexity (polynomial, non-polynomial (NP), NP-hard); the data certainty (deterministic or stochastic).

In a quantitative analysis of the optimization problems explored in the context of 6G RRM, the following observations emerge:

- Convexity: Approximately 30% of the optimization problems investigated exhibited convex behavior. This indicates that a significant portion of the research is focused on problems that are inherently solvable through convex optimization techniques, which are typically more straightforward and efficient due to their well-defined properties.
- Non-Convex to Convex Transformation: About 20% of the optimization problems were initially non-convex but were transformed into convex forms. This transformation is crucial for solving complex problems where direct convex formulations are not possible, highlighting the importance of techniques such as convex relaxation and transformation methods.
- Multi-Objective Functions: Roughly 26% of the optimization problems addressed in the surveyed works involve multi-objective functions. This reflects the complex nature of 6G RRM, where multiple performance metrics must be simultaneously optimized, often requiring sophisticated algorithms and trade-off analysis.
- Linear Structure: A total of 14% of the optimization problems were characterized by a linear structure. Linear optimization problems are typically simpler and more computationally tractable, suggesting that a portion of the research focuses on optimizing problems where linear approximations are sufficient or advantageous.

Table 11 offers an overview of the objective functions and constraints used in the optimization problems formulated for resource allocation in the 6G RRM papers analyzed in this study. This table summarizes the diverse approaches adopted by various studies, highlighting the specific goals and the constraints considered. It can be concluded that a significant portion (52%) of the works selected focus on maximizing the amount of data transmitted per unit of time. Among these 52% of works, specific optimization objectives were further broken down as follows:

- A total of 30% of the studies focused on optimizing the data rate. The data rate represents the instantaneous speed at which information is transferred, and its optimization is particularly important for applications requiring high-speed connections, such as ultra-high definition video streaming and real-time gaming.
- A total of 14% of the works dedicated themselves to optimizing the throughput, which is a measure of how much data is successfully delivered over the network in a given time period. Throughput optimization addresses not only speed but also reliability, making it important for maintaining consistent service quality in fluctuating network conditions.
- A total of 8% of the selected studies focused on maximizing the channel capacity, which is the theoretical upper bound of the data rate that a channel can support. Channel capacity optimization is critical for spectral efficiency, especially in dense urban environments where spectrum scarcity is a major challenge.

The focus on data rate (30%) over throughput (14%) suggests a priority on maximizing peak performance in ideal conditions. However, the comparatively smaller attention to throughput raises a potential gap. For real-world 6G use cases—such as autonomous vehicles or smart cities—reliable and consistent throughput is often more critical than peak data rates. Future research should consider shifting focus towards optimizing throughput in diverse and dynamic environments, where reliability is just as important as speed.

The relatively small proportion (8%) of works focused on channel capacity indicates that while channel efficiency is recognized, it may not yet be a dominant research focus in 6G RRM. However, as 6G networks push towards ultra-dense environments, CF architectures, and advanced beamforming techniques, optimizing channel capacity will become increasingly important.

The trends seen in the data suggest that current research efforts are largely centered around traditional RRM objectives (data rate, throughput, and channel capacity). However, 6G introduces new enabling technologies, such as mMIMO, RIS, and ISAC, which may require a shift in optimization priorities. For example, RIS can dynamically reconfigure the wireless environment, potentially altering the relationship between data rate and channel capacity. This creates opportunities for more holistic approaches to RRM optimization that consider the unique capabilities of these new technologies.

While the current focus on data transmission optimization is essential, it may overlook other critical aspects of RRM, such as energy efficiency, latency, and quality of experience. As 6G networks are expected to support a wide range of services with vastly different requirements—ranging from ultra-low latency applications to energy-constrained IoT devices—a more comprehensive approach to RRM optimization is necessary. Future research should explore multi-objective optimization frameworks that can balance competing priorities such as speed, energy consumption, and service reliability.

Continuing the analysis of Table 11 and focusing on the RRM function related to QoS management, 16% of the works on 6G RRM selected for this survey specifically investigated the time resource. Within these studies, the most frequently examined QoS parameters related to the time resource were average delay and latency. This emphasis on time-sensitive metrics suggests that research efforts are responding to the need for near-instantaneous data transmission.

As 6G aims to enable immersive experiences, holographic communications and remote surgeries, there are demands for further research on latency-sensitive optimization techniques, especially dealing with multi-objective optimization frameworks that consider the interplay between time resource optimization and other RRM functions. Technologies like network slicing and AI-based resource orchestration are expected to play a major role in improving the time resource management function in 6G. By allowing tailored QoS profiles for different services, network slicing can ensure that high-priority, latency-sensitive applications receive the necessary resources. Meanwhile, AI can be used to predict network conditions in real time and allocate resources accordingly, thereby minimizing delay and improving overall network performance. The integration of such advanced enablers is likely to elevate time resource optimization beyond traditional delay and latency metrics, creating more predictive and adaptive QoS solutions.

According to Table 11, 20% of the works on 6G RRM included in this survey investigated the energy resource, highlighting a growing concern for energy management in 6G networks. The 8% focus on maximizing energy efficiency reflects the shifting priorities in 6G toward greener and more sustainable communication systems. The 6% of studies focusing on minimizing transmission power illustrate the need to reduce interference while maintaining high system performance. Another 6% of the works explored minimizing overall energy consumption, which includes not just transmission but also aspects such as signal processing, idle mode energy and control signaling overhead. By considering total system energy consumption, these studies aim to optimize the entire network’s operation, not just individual links, making it a more holistic approach to energy management in 6G.

While energy efficiency is being addressed, the relatively low percentage of works focusing on energy resource optimization suggests that energy sustainability is still an underexplored area in 6G RRM research. As 6G pushes the boundaries of connectivity, with THz communication, RIS, and AI-driven resource management, energy optimization strategies will need to evolve. Future research could delve deeper into energy harvesting technologies, WPCN and the integration of energy-efficient hardware to further reduce the energy demands of 6G systems.

The studies focusing on energy resource management reveal that trade-offs between different objectives (e.g., energy efficiency vs. latency, data rate vs. power minimization) are becoming a critical aspect of 6G RRM optimization. For example, maximizing energy efficiency might sometimes come at the cost of lower data rates or increased latency, particularly in resource-constrained environments. This calls for multi-objective optimization frameworks that can balance these competing requirements dynamically, especially as 6G networks incorporate AI and ML to make real-time decisions about resource allocation and energy management.

Finally, one can note from Table 11 that only 12% of the works on 6G RRM selected for this survey explicitly investigated the frequency spectrum resource. This relatively small percentage reflects the complexity of managing the frequency spectrum in 6G networks and the potential gap in research. Within these studies, 10% of the objective functions in the optimization problems related to the frequency spectrum resource were specifically aimed at maximizing spectral efficiency. Thus, there are demands for further research on spectrum management considering both the TN-NTN integration and the multi-band spectrum use, as well as full-duplex and THz communication. Techniques such as AI-driven spectrum management, which leverage ML to predict spectrum availability and optimize spectrum usage, are likely to gain traction in 6G.

Table 12 provides an overview of the optimization strategies and algorithms used in the selected studies on 6G RRM, by exploiting a classification scheme developed in this survey. Such classification is described as follows:

- Combinatorial Optimization Methods: These techniques are used to tackle optimization problems where the solution involves selecting a discrete set of options from a finite or countable set. They are important for problems like user association and scheduling in RRM.
- Convex and Non-Convex Optimization Methods: Convex optimization methods are applied to problems where the objective function and constraints form a convex set, making them solvable with well-established techniques. Non-convex optimization methods are used for more complex scenarios where the problem landscape includes multiple local minima and requires advanced algorithms to navigate.
- Customized Algorithms for 6G RRM: Tailored algorithms designed specifically for 6G RRM functions address unique challenges such as dynamic resource allocation, interference management, and network slicing, reflecting the specific requirements and constraints of next-generation networks.
- Decomposition Methods: These techniques break down complex optimization problems into simpler subproblems, which can be solved more easily and efficiently. They are particularly useful for large-scale RRM problems where direct solution methods are impractical.
- Game Theory Methods: Game theory provides a framework for modeling and analyzing interactions among multiple agents or entities in RRM, such as network operators and users, to achieve optimal resource allocation and performance.
- Heuristic Methods: Heuristic approaches offer practical solutions by using rules of thumb or approximation strategies. They are useful for problems where exact solutions are computationally infeasible, such as dynamic user scheduling and load balancing.
- Machine Learning-Based Methods: Leveraging machine learning techniques, these methods learn from data to predict and optimize resource management strategies. They are increasingly used for tasks such as traffic prediction and adaptive resource allocation.
- Mathematical Programming Methods: These methods formulate optimization problems as mathematical models and solve them using algebraic techniques. They include linear programming, integer programming, and quadratic programming, which are used to handle various RRM functions.
- Metaheuristic Methods: These advanced optimization techniques, such as genetic algorithms and particle swarm optimization, are used to find approximate solutions to complex optimization problems, often involving multiple objectives and constraints.
- Stochastic Methods: Stochastic approaches incorporate randomness and probabilistic models to address uncertainties in optimization problems. They are useful in scenarios where network conditions are variable and uncertain.
- Transformation and Relaxation Methods for Non-Convex Problems: These methods convert non-convex problems into convex or easier-to-solve problems through relaxation or approximation techniques. They help in addressing the inherent complexity of non-convex optimization in RRM.

From the analysis of Table 12 regarding the methods employed to solve optimization problems, one can note that 57% of the selected works utilized some form of mathematical programming to make the problems more tractable. These mathematical programming methods often served as a precursor for further problem transformation or decomposition, highlighting the complexity of 6G RRM optimization issues, which typically require a multi-stage approach to make them solvable with reasonable computational effort.

Next in prevalence are the transformation and relaxation methods for non-convex problems, used in 30% of the selected papers. These methods were employed to convert non-convex problems into convex forms, which are easier to solve due to the well-understood properties of convex optimization, where local minima are also global minima. This suggests that a significant portion of 6G RRM problems are inherently non-convex, reinforcing the complexity of the optimization challenges in this field.

In 23% of the selected papers, convex and non-convex optimization methods were applied directly to solve the optimization problems. This indicates that even with the complexity and scale of 6G RRM issues, some problems are either naturally convex or can be tackled using more advanced techniques for non-convex optimization. Following this, 23% of the optimization problems were solved using metaheuristic methods, which provide flexible and powerful solutions to complex optimization problems that are otherwise too difficult to solve using exact methods.

When considering heuristic methods—used in 13% of the selected papers—the combined use of heuristic and metaheuristic methods accounts for 36% of the selected papers. This shows a clear trend in the use of adaptive, problem-specific algorithms that rely on experience-based techniques to explore the solution space efficiently. The widespread use of these methods highlights their importance in solving the highly complex and large-scale optimization problems prevalent in 6G RRM, where exact solutions may be computationally prohibitive.

Stochastic methods were applied in 18% of the selected papers to handle the inherent uncertainty and randomness in 6G RRM scenarios, such as varying network conditions or user behavior. Stochastic approaches are well-suited for such problems because they can model uncertainty and optimize under probabilistic constraints, making them valuable in the dynamic and unpredictable environments characteristic of future 6G networks.

Finally, ML-based methods were used in 30% of the selected papers. This reflects the growing trend of using ML and AI to optimize RRM functions, with methods such as reinforcement learning, deep learning, and supervised learning increasingly being applied to manage complex resource allocation, user association, and traffic scheduling tasks. These ML-based methods bring adaptability and the ability to learn from data, which is crucial in the context of 6G networks with their rapidly changing topologies and user requirements.

The dominance of mathematical programming methods (57%) emphasizes the need for structured approaches that can transform the original problem into a more manageable form. In many 6G RRM scenarios, problems are initially formulated as mixed-integer non-linear programs or involve other complex forms that require decomposition into sub-problems that can be solved more efficiently. This reflects a trend toward breaking down highly non-linear, multi-dimensional optimization tasks into more solvable units.

The frequent use of transformation and relaxation methods (30%) suggests that non-convexity remains a major hurdle in 6G optimization problems. These methods, which convert non-convex problems into convex forms, indicate that many real-world problems in 6G are not directly tractable with conventional optimization techniques. The ability to transform these problems into convex forms enables the use of more efficient, well-understood algorithms for solution, reducing computational overhead.

The significant reliance on heuristic and metaheuristic methods (36% combined) demonstrates the complexity and intractability of many 6G RRM problems, where exact solutions are impractical due to time constraints or problem scale. Metaheuristic algorithms such as genetic algorithms (GA), particle swarm optimization (PSO), and simulated annealing (SA) are widely favored because they offer flexibility, scalability, and the ability to find near-optimal solutions for problems that traditional methods struggle with. These methods can efficiently navigate large solution spaces and handle multi-objective optimization, which is important in the context of 6G.

Meanwhile, the use of ML-based methods (30%) points to the future of 6G RRM, where networks will need to self-optimize based on real-time data. ML models, particularly in reinforcement learning and deep neural networks, will play a pivotal role in enabling networks to learn and adapt, providing more efficient, autonomous RRM decisions over time.

The use of AI/ML has gained significant importance in recent works on RRM. Among the fourteen most recent articles referenced in this paper, eleven consider the use of AI/ML in some form. The main approaches utilized are: reinforcement learning, neural networks and graph models, explainable AI (XAI), and heuristic and meta-heuristic methods. Below is a summary of the main techniques used in these references:

- Multi-Agent Reinforcement Learning (MARL): An offline approach of this technique is used in [94]. Core techniques include the Conservative Q-Learning (CQL) algorithm, used to handle uncertainty and avoid overestimation in static data, and the Soft Actor-Critic (SAC) framework for training stability. The Centralized Training Decentralized Execution (CTDE) architecture is applied, combined with Value Decomposition methods to approximate the global value function. Paper [107] also utilizes this technique, with agents based on deep Q-networks (DQN) distributed across vehicles.
- Deep Reinforcement Learning (DRL): Paper [132] mentions the use of DQN for network slicing and MAC layer scheduling, while ref. [108] uses the same algorithm for resource management in network slices. Paper [188] uses DQN integrated with Graph Neural Networks (GNN), such as Graph Convolutional Networks (GCN) and Graph Attention Networks (GAT), to encode network topology and cell attributes. Paper [26] highlights the utilization of DRL with DQN to optimize dynamic spectrum access (DSA).
- Explainable AI (XAI): Paper [108] uses the EXPLORA framework to generate network-oriented explanations. The Attributed Graph Models technique is employed to link agent actions to input states within the O-RAN architecture. Paper [107] also uses XAI techniques based on the SHAP (Shapley Additive Explanations) and Deep-SHAP methods to perform feature selection and reduce model complexity.
- Offline and Distributional RL: Paper [96] utilizes the CQL algorithm to handle static data and Quantile-Regression DQN (QR-DQN) to estimate the distribution of returns in stochastic environments. The proposed technique, Conservative Quantile Regression (CQR), merges these two approaches.
- Unsupervised Machine Learning: Paper [103] utilizes this approach in its framework.
- Graph Neural Networks and Temporal Graph Neural Networks (TGNN): Paper [90] proposes a TGNN that operates on Continuous-Time Dynamic Graphs (CTDG). The model uses Gated Recurrent Units (GRU) to maintain node-level and graph-level memories. Training combines supervised learning (using data generated by genetic algorithms as labels) and unsupervised learning for fine-tuning during deployment. Paper [26] presents the use of GNNs to model complex interactions, in addition to exploring the use of federated learning (FL) techniques for privacy preservation.
- Radial Basis Function Network (RBFN): Paper [91] combines RL (including algorithms such as Q-learning, SARSA, and CACLA) for scheduling decisions with a Radial Basis Function Network (RBFN) for pattern classification.
- Meta-heuristic methods: Paper [91] employs a meta-heuristic based on simulated annealing with stochastic tunneling (SAST) to optimize data clustering and neural network training. Additionally, heuristics such as D-MADOC, H-RRM, and D-MCC-DSatur utilize user location and graph coloring techniques (DSatur algorithm) for MU-MIMO scheduling and co-channel interference reduction [95].

## 6. Conclusions

6G networks represent the next evolution in wireless communications technology, designed to build on and surpass the capabilities of 5G. They are expected to serve a wide range of applications and services that require extremely high data rates, ultra-low latency, massive connectivity, and increased reliability. The goal of 6G is to create a smart, widespread, and secure network that integrates the physical, digital, and biological worlds.

6G networks must support high connection density and facilitate the movement of large amounts of data between different entities, including humans, machines, and human–machine interactions. They are expected to provide ubiquitous global coverage through terrestrial and non-terrestrial network integration and sensing techniques. The extensive use of AI in a context that includes cell-free, massive MIMO, and beamforming technologies is essential. Moreover, 6G networks must be human-centered, aligning with ambitious global projects like the UN’s 2030 SDGs, and addressing the social, economic, and environmental needs of modern society.

RRM is fundamental to the design and efficient operation of 6G networks. By dynamically allocating, scheduling, and optimizing radio resources, RRM ensures high performance, QoS, and efficient use of scarce natural resources. As 6G networks aim to deliver ultra-high data rates, ultra-low latency, and massive connectivity, RRM becomes indispensable in achieving these advanced characteristics and ambitious goals.

The significance of RRM in 6G lies in its ability to handle ultra-high speeds and low-latency demands, manage massive connectivity, and ensure dynamic spectrum sharing. RRM’s role in network slicing allows for tailored resource allocation to meet diverse service requirements. Additionally, integrating AI into RRM systems enables real-time decision-making, traffic pattern prediction, and resource optimization, ensuring adaptive and efficient network performance in highly dynamic environments.

Specific techniques like NOMA, OFDMA, and OTFS highlight the advanced resource allocation strategies important for 6G. Moreover, RRM supports energy efficiency through power control and energy-aware strategies, maintains connectivity through seamless mobility management, and enhances signal quality with massive MIMO and beamforming. These capabilities underscore RRM’s critical role in realizing the vision of 6G networks, marked by unparalleled speed, connectivity, and intelligence.

In summary, the optimization of scheduling, resource allocation, and the use of radio resources is crucial for the efficient operation of 6G networks. Addressing the challenges posed by 6G’s ambitious KPIs, such as ultra-low latency, high data rates, massive connectivity, and enhanced reliability, requires advanced optimization methods and algorithms. Techniques like linear and non-linear programming, heuristic and metaheuristic algorithms, game theory, and convex optimization are integral to solving complex resource allocation problems. Additionally, the integration of AI and ML, including reinforcement learning and deep learning, enables real-time decision-making, predictive analysis, and adaptive optimization, ensuring dynamic and efficient network performance.

Key challenges in 6G optimization include accurate CSI and sensing, efficient resource management in cell-free architectures, seamless TN-NTN integration, and the coordination of 3D heterogeneous networks. Advanced interference mitigation and control strategies are essential to minimize co-channel and inter-cell interference in densely populated network environments. Furthermore, meeting the high demands of massive device connections and high data traffic necessitates scalable and robust optimization algorithms that can operate effectively in real time.

Looking ahead, the trends in 6G optimization focus on incorporating security and privacy constraints into optimization methods to protect data integrity while maintaining performance. Sustainability is another critical aspect, driving the need for energy-efficient optimization techniques to reduce the carbon footprint of 6G networks. Service differentiation through precise resource allocation and tailored network slices will be crucial in addressing the diverse requirements of 6G applications. Continuous research and development in optimization techniques will be important to fully realize the transformative potential of 6G networks, ensuring a seamless integration of the physical, digital, and biological worlds.

In addition to technical advancements, ensuring robust security measures and promoting sustainability are critical for the long-term success of 6G networks. The deployment of 6G will support a wide range of innovative services, from immersive experiences to smart city infrastructure and remote healthcare. As we move towards a hyper-connected world, 6G will play a pivotal role in shaping the future of wireless communication, driving societal and technological advancements, and enabling new possibilities in our digital landscape.

This paper presented a detailed survey on RRM in 6G CF mMIMO networks, addressing the various aspects that impact the performance and applicability of RRM algorithms, as well as identifying opportunities for further research.

## Figures and Tables

**Table 2:** Summary of KPIs for 5G and 6G considered in related works.

KPI	5G	6G
Peak data rate	20 (Gb/s)	50–200 (Gb/s) [5]; >100 (Gb/s) [59]; 1 (Tb/s) [32,60,61,62,63]; >1 (Tb/s) [29,64,65]
User experienced data rate	100 (Mb/s)	300–500 (Mb/s) [5]; 1 (Gb/s) [65]; 10 (Gb/s) [63]
Spectrum efficiency	0.3 (b/s/Hz)	1.5–3× relative to 5G [5]; >3× relative to 5G [29]
Area traffic capacity	10 (Mb/s/m^2^)	30–50 (Mb/s/m^2^) [5]; >100 (Mb/s/m^2^) [29,59]; 1–10 (Gb/s/m^3^) [63]; 1 (Gb/s/m^2^) [65]
Connection density	1 × 10^6^ (dev/km^2^)	10^6^–10^8^ (dev/km^2^) [5]; 10^7^ (dev/km^2^) [65]; 10^8^ (dev/km^2^) [32]
Mobility	500 (km/h)	500–1000 (km/h) [5]; 1000 km/h) [64]; >1000 (km/h) [29,59,65]
Latency	1 (ms)	0.1 (ms) [32,60,63,64]; 0.1–1.0 (ms) [5,65]; <1 (ms) [29,59,61,62,66]
Reliability	99.999 (%)	>99.999 (%) [59]; 99.9999 (%) [60]; 99.999–99.99999 (%) [5]; 99.99999 (%) [29]; 99.9999999 (%) [32]
Positioning accuracy	10 (cm 2D)	1–10 (cm) [5]; 1 (cm) [32]; 1 (cm in 3D) [63]; 10 (cm) indoor and 1 (m) outdoor [60]
Energy efficiency	Not specified	10× relative to 5G [29,32]; 1 (pJ/bit) [63]
Frequency band	3–300 (GHz)	T (Hz) [60,61,62,63,65]
Receiver sensitivity	−120 (dBm)	<−130 (dBm) [29]

**Table 3:** Summary of multiple access techniques examined in works on RRM.

Multiplexing Technique	Type/Domain	Related Works
OMA	OFDMA	[34,39,41,42,44,45,46,49,73,81,83,84,85,86,87,88,89,90,91]
	OTFS	[92]
	FDMA—Frequency Division Multiple Access	[33,93]
	TDMA—Time Division Multiple Access	[33,70,94,95,96]
	D-OMA	[76]
NOMA	Power	[33,40,48,69,75,76,80,86,93,97,98]
	Space	[48,80,93,97]
	Hybrid	[35,50]
	RSMA—Rate-Splitting Multiple Access	[43]
	Cooperative	[69]
	AS/SDA	[37]

**Table 4:** Summary of the 6G network geographical arrangements.

Generation	Infrastructure	Network	Tiers	Related Works
5G	Cellular	2D HomNet	TN	[33,103]
6G	Cellular	2D HetNet	TN	[34,40,48,49,69,71,75,83,84,88,91,94,96,104,105,106,107,108]
6G	Cellular	3D HetNet	TN + NTN	[35,39,51,70,81,87,89,93,95,97,109,110,111]
6G	Cell-Free	2D HetNet	TN	[112,113,114]
6G	Cell-Free	3D HetNet	TN + NTN	[36,90]
B5G/6G	Cellular	2D HetNet	TN	[41,47,115]
B5G/6G	Cellular	2D HomNet	TN	[42,45,46,92]
6G	Cellular	2D HomNet	TN	[37,38,43,44,50,72,73,77,80,86,98,116,117,118,119]
6G	Cell-Less	2D HetNet	TN	[76,85]

**Table 5:** Infrastructure considered in 6G RRM studies.

Infrastructure	RAN Type	Related Works
Communication Mode	Terrestrial Base Station	[34,35,37,39,40,41,42,44,45,46,47,50,51,69,70,71,72,75,76,77,83,86,87,88,90,92,98,105,106,107,109,110,113,116,117,119,121,131,132]
	Access Point	[33,38,48,70,73,75,84,85,94,96,109,112,113,114]
	GEO	[43,109]
	LEO	[35,39,51,81,89,97,109]
	VLEO	[95]
	HAPS	[51]
	UAV	[51,70,87,90,93,94,97,110,111]
	D2D	[90,110]
	Relay Node	[33,37,51,70,83,93,104,105,110,111]

**Table 6:** Allocated resources in 6G RRM studies.

Resource Area	Assigned Resources	Related Works
Power		[33,34,35,38,39,40,43,44,45,46,49,69,70,71,73,75,76,77,80,89,90,93,97,98,104,107,109,110,113,117,118,119]
	Energy (J/Wh)	[37,69,70,72,111]
Frequency	Resource Block (RB)	[39,42,43,44,45,46,48,49,50,71,76,83,84,85,86,87,90,91,92,93,103,108]
	BW/Channel	[33,36,40,41,46,47,51,70,73,75,81,89,90,93,96,104,105,106,107,109,112,113,116,118,131]
Scheduling	Time/Time Slot	[33,37,39,40,41,42,45,46,48,49,50,70,71,73,76,83,84,85,86,87,104,106,109,111,114]
Antenna	Beamforming	[43,77,80,81,83,87,89,92,93,95,105,114,117,119,131]
	RIS	[73,105,110]
Computing	Processing	[44,47,48,71,72,73,98,104,112,116]
	Caching/Memory	[35,47,48,71,93,109,116]
	Slicing	[41,44,45,46,71,103,108]
	Edge Computing	[47,73,98,109,116]
	Cloud Computing	[71,98]

**Table 7:** Summary of resource allocation and scheduling in RRM research.

RRM Type	Direction	I/O	Related Works
RA	DL	SISO	[35,38,43,46,47,51,69,72,75,76,89,98,106,107,111,116,118]
		MIMO	[92,93,97,117,131]
		mMIMO	[112,119]
	UL	SISO	[44,104,105,107,110]
		MIMO	[34,131]
		mMIMO	[36,80,112,113]
RA + SCH	DL	SISO	[39,40,42,71,81,86,94,109]
		MIMO	[37,45,77,83,95,114]
		mMIMO	[85,88]
	UL	SISO	[33,41,49,50,70,73,81,105]
		SIMO	[84]
		MIMO	[87]
		mMIMO	[48]

**Table 8:** Spatial resource allocation techniques in 6G RRM.

Resource Area	Techniques	Related Works
Space	Diversity	[43,48,69,80,81,83,84,88,92,93,95,97,114]
	Sensing	[70,87,92,97,117,118,119]
	Cluster	[36,43,48,51,76,77,80,81,84,86,91,93,95,103,106,111,112,114]
	Handoff	[34,51]
	Interference mitigation	[34,36,38,43,44,70,81,85,88,89,92,94,95,96,105,107,109,110,112,114,117]
	SIC	[33,35,37,40,48,50,69,75,76,80,86,93,97,104,114]

**Table 9:** Access and communication resource management summary.

Access and Communication Resource Management	Related Works
User Association	[34,35,39,43,51,70,80,85,89,93,94,96,106,112,131]
Communication Mode Selection	[33,36,51,69,75,76,77,84,87,97,105,107,109,112,114,131]
Admission Control	[33,40,45,47,48,86]

**Table 10:** Key technologies and techniques utilized in 6G RRM studies.

Key Technology	Related Works
3D HetNet	[35,36,39,43,51,69,81,87,89,90,95,109,110,111]
AI	[26,35,38,41,47,48,50,51,84,85,86,90,91,94,95,96,98,103,105,106,107,108,116,132,188]
Beamforming	[43,77,80,81,83,89,92,93,95,105,110,114,117,119,131]
Cell-Free	[36,48,90,112,113,114]
Cell-Less	[75,76,85]
Cluster	[36,43,48,51,76,77,80,81,84,86,91,93,95,103,106,111,112,114]
Combiner	[36,48,50,80,90,112,113,114]
CoMP	[39,69,76]
D2D	[90,110]
Diversity	[40,43,48,50,70,76,80,81,83,84,86,92,93,97,98,114]
Edge Computing	[47,48,71,73,84,98,103,108,109,116]
Energy Harvesting, WPT, WIT, WPCN	[33,37,49,70,111,114]
Ensemble Strategy	[75]
Finite Blocklength	[49]
IoT	[33,34,35,36,37,38,39,40,90,114]
mMIMO, MIMO	[37,39,48,77,80,88,92,93,95,97,105,112,113,114,131]
NOMA	[33,35,37,40,48,50,69,75,76,80,86,93,97,98,114]
NTN	[35,39,43,51,70,81,87,89,90,93,95,97,109]
Numerology	[45,50,87]
O-RAN, Flexible RAN, C-RAN, F-RAN, NG-RAN	[44,45,47,48,51,71,77,81,85,98,103,108,112,116,132]
Precoding Methods	[48,77,83,88,90,95,97,112]
Puncturing	[41,42,45,46,103]
QoE	[41,76,87,108]
Quantum Computing	[39,83,98]
Relay Node	[33,37,51,70,93,104,110,111]
RIS	[73,83,105,110]
Semantic	[72,106]
Sensing, ISAC	[70,87,92,97,117,118,119]
SIC	[33,35,37,40,48,50,69,76,80,86,93,97,104,114]
Slicing	[41,44,45,46,48,71,103,108]
THz, mmWave	[103,110]
TTI	[42,45,50,51,83,91,108]

**Table 11:** Optimization problems for 6G RRM: objective functions and constraints.

Objective	Constraints and Related Works
Max Energy Efficiency	Min Rate [69,70,71]; Max Power [35,69,70]; Max Energy [70]; Max Time [71]; Queue Stability [71]; Interference, CSI, SINR [70]; Computer Load [71]; Cache Memory [35,71]; Number of Links [35]; Number of Users [35,70].
Min Power	Min Rate [69]; Max Power [46,69,73]; Max Delay [46,73]; Queue Stability [46]; Angle/Phase [73]; Computer Load [73]; Number of Links [46]; Prob. Outage [73].
Min Energy	Min Rate [37,44]; Max Power [44,72]; Max Energy [37]; Max BW [44]; Max Delay [72]; Max Latency [44]; Max Time [37]; Capacity [44]; Computer Load [44]; Amount of Links [44]; Semantics [72].
Max SE	Min Rate [41,80]; Max Power [43,77,80]; Max BW [41]; Max Latency [41]; Interference, CSI, SINR [36,80]; Sparsity [77].
Max Success Probability	Min Rate [76,93]; Max Power [49,84]; Max Latency [49,50]; Max Time [84]; Capacity [49]; Interference, CSI, SINR [49]; Amount of Links [76]; Reliability [50]; Sparsity [50].
Max Rate	Min Rate [51,69,86,98,113]; Max Power [33,38,43,45,69,86,92,97,98,107,110,113]; Max Energy [33]; Max Distance [93]; Max BW [45,51,105]; Max Latency [45,88]; Interference, CSI, SINR [36,97,105]; Angle/Phase [105,110]; Number of Links [51,86,105]; Number of Users [93]; Prob. Outage [38]; Reliability [45,88,107]; Min Distance [94,96]; Min Throughput [90]; Fixed Power [96]; Max BLER (Block Error Ratio) [88].
Max Capacity	Min Rate [85,109]; Max Power [95,109,118]; Max BW [81]; Number of Links [81,85,109]; Location Accuracy [118]; Number of Clusters [95].
Max Throughput	Min Rate [40,42,131]; Max Power [39,40,75,89,114]; Max BW [42,106,112,131]; Queue Stability [39]; Computer Load [112]; Number of Links [39,106,131]; Number of Users [39,40,131]; Prob. Outage [42]; Energy Consumption [114]; Min Primary Throughput [114]
Min Time or Max Time	Min Rate [83,87,111]; Max Power [116]; Max Energy [111]; Max Distance [111]; Max BW [83,87]; Max Latency [116]; Max Time [87]; Capacity [87]; Computer Load [116]; Cache Memory [116]; Number of Links [87].
Min Delay	Min Rate [71]; Max Power [93,104]; Max Energy [48]; Max Distance [93]; Max BW [48,104]; Max Time [48,71]; Queue Stability [71,104]; Interference, CSI, SINR [93]; Computer Load [48,71]; Cache Memory [48,71,93]; Amount of Links [48]; Number of Users [104].
Max QoE or Max QoS	Min Rate [41,91]; Max Power [76]; Max BW [41]; Max Latency [41,91]; Number of Links [76]; Packet Loss [91].
Min Inteference	Max Power [34]; Max Distance [34]; Interference, CSI, SINR [34].
Max Users	Min Rate [87,111]; Max Energy [111]; Max Distance [111]; Max BW [87,106]; Max Time [87]; Capacity [87]; Amount of Links [87,106]; Cloud Computing [132].
Max Security	Min Rate [119]; Max Power [117,119]; Max Time [119]; Angle/Phase [119]; Location Accuracy [117]; Security [119].
Max Profity	Min Rate [47]; Max Time [47]; Computer Load [47].
Min Distance	Min Rate [111]; Max Energy [111]; Max Distance [111].
Min BW	Max Power [46]; Max Delay [46]; Queue Stability [46]; Number of Links [46]; Total number of PRBs [103].
Min Cost	Min Rate [44,86]; Max Power [44,86]; Max BW [44]; Max Latency [44]; Capacity [44]; Computer Load [44]; Number of Links [44,86]
Min Handoff	Min Rate [51]; Max BW [51]; Number of Links [51].

**Table 12:** Overview and classification of 6G RRM optimization approaches.

Classification	Proposed Solutions and Related Works
Combinatorial Optimization Methods	Branch and Bound (BnB) [51]; Knapsack Problem [42]; Kuhn-Munkres Algorithm [33,104]; List-Processing Algorithm [112]; Quadratic Unconstrained Binary Optimization [39]
Combinatorial Optimization Method; Game Theory Method	One-to-One Matching Approach and Matching Theory [46,86]
Customized Algorithm for 6G RRM	Customized Algorithms [34,36,45,72,77,80,81]
Convex and Non-Convex Optimization Method	Frank-Wolfe Policy [70]; Sub-Gradient Method [37]; Standard Framework [89].
Convex and Non-Convex Optimization Method; Transformation and Relaxation Methods for Non-Convex Problems	Majorization Minimization Approximation Method [44]; Sequential Quadratic Method and Conic Quadratic Representation Method [37,86]; Successive Convex Approximation (SCA) [40,49,114]; Water-Filling Method [118]; Block Coordinate Descent (BCD) [114]; Semi-Definite Relaxation (SDR) [114]
Decomposition Method	Message Passing Algorithm [131]; Functional Decomposition [89]; Value Decomposition [94].
Decomposition Method; Mathematical Programming Method	BCD Algorithm [46,105,118,119]
Decomposition Method; Quantum Computing Method	Hybrid Quantum-Classical Generalized Benders’ Decomposition Algorithm [39]
Game Theory Method	Coalition Game Algorithm [105]; Shapley Additive Explanations [107].
Heuristic Methods	Golden Search Algorithm [118]; Greedy Method [40]; Proportional Fairness in Time and Frequency (PFTF) [42]
Heuristic Method; Stochastic Method	Maximum-Largest Weighted Delay First (M-LWDF) [42]
Machine Learning-Based Methods	Deep Neural Network (DNN) [113]; Deep Learning, Deep Reinforcement Learning, Reinforcement Learning Algorithms, and Deep Deterministic Policy Gradient [35,48,84,91,94,96,107,108,113]; Dueling Deep Q-Network (DQN), Double DQN, and Deep Q-Learning [38,41,50,116]; Temporal Graph Neural Network (TGNN) [90]; Unsupervised Machine Learning [103]; Radial Basis Function Network (RBFN) [91].
Machine Learning-Based Method; Heuristic Method	K-Means Algorithm [93,103,111]
Machine Learning-Based Method; Quantum Computing Method; Stochastic Method	Quantum Neural Networks [98]
Mathematical Programming Methods	Bisection Method [33,77]; Dinkelbach’s Algorithm [69,70]; Fractional Programming [43,69,71,110]; Optimization Solver, Linear Programming (LP) Relaxation, One-dimensional (1D) Linear Programming, and Gurobi Solver [49,51,70,87,92,109]; Primal-Dual Interior Point Method [69]; Mixed-Integer Nonlinear Programming [90,107].
Mathematical Programming Method; Convex and Non-Convex Optimization Method	Interior-Point Method (Barrier Method) [38,73,106]; Karush-Kuhn-Tucker Conditions [43,71,73,109,118]
Mathematical Programming Method; Decomposition Method	Schur Complement Technique [117]
Mathematical Programming Method; Stochastic Method	Stochastic Programming Model [106]
Mathematical Programming Method; Transformation and Relaxation Methods for Non-Convex Problems	Inner Approximation [119]
Metaheuristic Methods	Bi-Objective Selection Hyper-Heuristic (SHH) [76]; Genetic Algorithm, CAM-ADX, and NSGA-II [47,51,75,85,93,111]; Particle Swarm Optimization (PSO), SAPSO, SBPSO, DSBPSO, and PSO-NGDP [47,75,93,111,114]; Simulated Annealing [91]; D-MADOC, D-MCC-DSATUR and H-RRM [95].
Metaheuristic Methods; Heuristic Methods	Local Discrete Phase Search, Local Search, and Population-Based Algorithms [81,105]
Metaheuristic Method; Stochastic Method	Circular-Based Recursive Random Search (CRRS) [70]
Quantum Computing Method	Grover’s Quantum Search Algorithm [83]; Quantum Annealer [39]
Stochastic Method	Drift-Plus-Penalty [73,86]; Markov Inequality [45,46]; Markov Decision Process [103].
Stochastic Method; Game Theory Method	Markov Decision Process [116]
Stochastic Method; Mathematical Programming Method	Lyapunov Optimization and Lyapunov Stochastic Optimization [46,71,73,86,104]
Transformation and Relaxation Methods for Non-Convex Problems	Charnes-Cooper Transformation [117]; First-Order Taylor Expansion and Taylor Approximation [44,97]; Lagrange Multiplier and Lagrange Duality Method [33,39,43,77,104,109,110,117]; Normal Convex Relaxation Method and SDR Method [45,77,117,119]; Quadratic Transform (QT) Algorithm [80]

## Data Availability

The original contributions presented in this study are included in the article. Further inquiries can be directed to the corresponding author.

## Abbreviations

1D	One-dimensional
2D	Two-dimensional
3D	Three-dimensional
3G	Third Generation of cellular network
3GPP	3rd Generation Partnership Project
4G	Fourth Generation of cellular network
5G	Fifth Generation of cellular network
6G	Sixth Generation of cellular network
AI	Artificial Intelligence
AmBC	Ambient backscatter communication
AoA	Angle of Arrival
AoD	Angle of Departure (AoD)
AP	Access Point
B5G	Beyond 5G
BBM	Beam and Beamforming Management
BCD	Block Coordinate Descent
BS	Base Station
CAM-ADX	Computer-Aided Manufacturing-Automatic Deployment eXchange
CF	Cell-Free
CL	Cell-Less
CO_2_	Carbon Dioxide
CoMP	Coordinated Multi-Point
COVID	Coronavirus Disease
CP	Cyclic Prefix
CPU	Central Processing Unit
CQL	Conservative Q-Learning
C-RAN	Cloud Radio Access Networks
CRN	Cognitive Radio Network
CRRS	Circular-Based Recursive Random Search
CSI	Channel State Information
CTDE	Centralized Training Decentralized Execution
CTDG	Continuous-Time Dynamic Graphs
D2D	Device-to-Device (D2D)
DL	Downlink
DLT	Distributed Ledger Technologies
D-OMA	Delta-OMA
DQN	Deep Q-Network
DRAPS	Dynamic Resource Allocation and Puncturing Strategy
DRL	Deep Reinforcement Learning
DSA	Dynamic Spectrum Access
DSBPSO	Dual Stage Binary Particle Swarm Optimization
DSM	Dynamic Spectrum Management
EE	Energy Efficiency
eMBB	Enhanced Mobile Broadband
ETSI	European Telecommunications Standards Institute
FBMC	Filter Bank Multicarrier
FDMA	Frequency Division Multiple Access
FL	Federated Learning
FT-QNN	Federated Quantum Neural Network (FT-QNN)
GA	Genetic Algorithm
GAT	Graph Attention Networks
GCN	Graph Convolutional Networks
GEO	Geostationary Earth Orbit
GF	Grant-Free
GNN	Graph Neural Networks
GRU	Gated Recurrent Units
GSMA	GSM Association
HAP	High Altitude Platform (HAP)
HetNet	Heterogeneous Network
ICT	Information and Communication Technology
IMT	International Mobile Telecommunications
IoT	Internet of Things
IoTDs	IoT devices
IRS	Intelligent Reflecting Surface
ISAC	Integrated Sensing and Communication
ISI	Intersymbol Interference
ITU	International Telecommunication Union
KPI	Key Performance Indicator
LEO	Low Earth Orbit
MARL	Multi-Agent Reinforcement Learning
MC	Multi-Carrier
MCG	Multiple Configured-grants
MEC	Mobile Edge Computing
MEO	Medium Earth Orbit
ML	Machine Learning
mMIMO	massive Multiple-Input Multiple-Output
mMTC	massive Machine Type Communication
mmWave	millimeter-wave
NGDP	Next Generation Digital Platform
NOMA	Non-Orthogonal Multiple Access
NP	Non-Polynomial
NSGA	Non-dominated Sorting Genetic Algorithm
NTN	Non-Terrestrial Network
OFDM	Orthogonal Frequency Division Multiplexing
OFDMA	Orthogonal Frequency Division Multiple Access
OMA	Orthogonal Multiple Access
OOBE	Out-of-Band Emission
OTFS	Orthogonal Time Frequency Space
PBAS	Pilot-Based Access Point Selection
PC&A	Power Control and Power Allocation
PSO	Particle Swarm Optimization
QoS	Quality of Service
QoE	Quality of Experience
QR-DQN	Quantile-Regression DQN
RAN	Radio Access Network
RAS	Robotics and Autonomous Systems
RF	Radiofrequency
RIS	Reconfigurable Intelligent Surface
RL	Reinforcement Learning
RRM	Radio Resource Management
RSMA	Rate-splitting Multiple Access
SA	Simulated Annealing
SAC	Soft Actor-Critic
SAPSO	Self-Adaptive Particle Swarm Optimization
SBPSO	Subband Binary Particle Swarm Optimization
SCMA	Sparse Code Multiple Access
SDG	Sustainable Development Goals
SDR	Semi-Definite Relaxation
SE	Spectral Efficiency
SIC	Successive Interference Cancellation
SINR	Signal-to-Interference-plus-Noise Ratio
SON	Self-Organizing Network
SSM	Smart Spectrum Management
SWIPT	Simultaneous Wireless Information and Power Transfer
TDMA	Time Division Multiple Access
TGNN	Temporal Graph Neural Networks
THz	Terahertz
TN	Terrestrial Network
UAV	Unmanned Aerial Vehicle
UE	User Equipment
UL	Uplink
uRLLC	ultra-Reliable Low-Latency Communication
WPCN	Wireless Powered Communication Network
WPT	Wireless Power Transfer
XAI	Explainable AI
